# miR-146a-5p-modified hUCMSC-derived exosomes facilitate spinal cord function recovery by targeting neurotoxic astrocytes

**DOI:** 10.1186/s13287-022-03116-3

**Published:** 2022-09-30

**Authors:** Xunwei Lai, Yang Wang, Xiaokang wang, Bin Liu, Limin Rong

**Affiliations:** 1 Department of Spine Surgery, The Third Affiliated Hospital of Sun Yat-sen University, No. 600 Tianhe Road, Tianhe District, Guangzhou, Guangdong Province China; 2 National Medical Products Administration (NMPA) Key Laboratory for Quality Research and Evaluation of Cell Products, No. 600 Tianhe Road, Tianhe District, Guangzhou, Guangdong Province China; 3 Guangdong Provincial Center for Engineering and Technology Research of Minimally Invasive Spine Surgery, No. 600 Tianhe Road, Tianhe District, Guangzhou, Guangdong Province China; 4 Guangdong Provincial Center for Quality Control of Minimally Invasive Spine Surgery, No. 600 Tianhe Road, Tianhe District, Guangzhou, Guangdong Province China

**Keywords:** Modified exosomes, Neurotoxic astrocytes, Spinal cord injury, miR-146a-5p/Traf6/Irak1 axis

## Abstract

**Background:**

Acute spinal cord injury (SCI) is a devastating result of neurological trauma with subsequent microenvironment dyshomeostasis that induces neurotoxic phenotype acquisition by astrocytes, exacerbating neurological function impairment. Exosomes derived from human umbilical cord mesenchymal stem cells (hUCMSCs) have demonstrated essential therapeutic effects after central nervous system trauma. However, whether hUCMSC-derived exosomes exert therapeutic effects on neurotoxic astrocytes to facilitate SCI function recovery remains unclear. Additionally, the limited efficiency of single exosomes may restrict the optimization of exosomal biological functions.

**Methods:**

We first determined that exosomes reduce the deleterious effects of neurotoxic astrocytes in vitro and in vivo. Then, we identified critical functional microRNAs (miRNAs). miR-146a-5p was overexpressed in exosomes, and then, miR-146a-5p-modified exosomes were used to investigate the ability of exosomes to reduce neurotoxic astrocyte effects, preserve neurons and promote neurological function recovery in rats with SCI.

**Results:**

Cell counting kit-8 and neurite length analyses revealed that exosomes partially reduced the negative effects of neurotoxic astrocytes on PC12 cell viability and neurites in vitro. The exosomes also attenuated inflammatory responses, reduced the number of neurotoxic astrocytes and preserved neural tissue in rats with SCI. Immunofluorescence assays suggested that the number of neurotoxic astrocytes was rapidly increased by injury, reaching a peak 5 days post-injury (dpi) and returning to the normal level 14dpi. Exosomal miR-146a-5p was identified as the critical functional miRNA. Overexpression of miR-146a-5p in exosomes strengthened the biological function of the exosomes. Therefore, the modified exosomes exerted more powerful therapeutic effects than the unmodified exosomes, reducing the deleterious effects of neurotoxic astrocytes both in vitro and in vivo and promoting locomotor function of the hindlimbs in the rats with SCI. Through a series of gain- and loss-of-function experiments, Traf6 and Irak1 were identified as targets of exosomal miR-146a-5p. Ultimately, we found that miR-146a-5p-modified exosomes exerted their function by targeting Traf6/Irak1/NFκB pathway in neurotoxic astrocytes.

**Conclusions:**

In summary, miR-146a-5p-modified exosomes exerted a more powerful effect than unmodified exosomes to promote neurological function recovery in rats with SCI by targeting neurotoxic astrocytes. Therefore, miR-146a-5p-modified exosomes are promising therapeutics for SCI.

**Supplementary Information:**

The online version contains supplementary material available at 10.1186/s13287-022-03116-3.

## Introduction

Acute spinal cord injury (SCI) is a devastating neurological outcome of trauma resulting in dysfunction of sensory, motor or autonomic nerves [1]. Acute SCI consists of two basic pathophysiology phases. The primary phase involves immediate effects of the initial trauma, which typically require surgical intervention, and the second phase is the subsequent spinal microenvironment dyshomeostasis involving an inflammation cascade, ischemia and cytotoxic substance production, which aggravates damage to neural tissue [2–4].

Astrocytes are the most abundant cells in the healthy central nervous system (CNS) and exert many critical functions, including neurotrophic, blood flow regulation and other homeostatic maintenance functions [5]. Acute SCI triggers a serious inflammatory response involving microglial activation and immune cell infiltration, subsequently driving astrocyte reactivity [6]. Reactive astrocytes may exert neural preservation by contributing to tissue debris clearance and inhibiting excessive inflammatory responses [7, 8]. However, CNS insults also induce acquisition of the neurotoxic phenotype by astrocytes, causing corresponding excessive damage to neural tissue. Neurotoxic astrocytes lose their normal functions and exhibit neurotoxic effects on neurons and mature oligodendrocytes [9]. Therefore, reversing neurotoxic phenotype acquisition or eliminating the deleterious effects of neurotoxic astrocytes is an essential way to promote neurological recovery in SCI patients.

Exosomes are nanosized cellular vesicles with diameters ranging from 50 to 150 nm and that contain RNAs, DNAs and proteins inside the bilayer lipid membrane [10]. According to the literatures, stem cell-derived-exosomes share the biological effects similar to those of stem cells in inhibiting activation of microglia and relieving inflammation responses [11, 12]. However, some factors, such as a relatively low yield of exosomes from cell culture supernatant, limit exosome applications [13]. Interestingly, exosomes also possess advantages such as the capability to carry and protect nucleic acids, cross biological barriers, and target cells, qualifying them as excellent drug delivery systems[14]. Therapeutic RNAs such as small interfering RNA (siRNA) and microRNA (miRNA) can be loaded into exosomes to obtain better therapeutic effects [15, 16].

The preliminary results of our study demonstrated that human umbilical cord mesenchymal stem cell (hUCMSC)-derived exosomes (MSC-Exos) partially reduced the deleterious effects of neurotoxic astrocytes to preserve neurons and neural tissue in vitro and in vivo. Subsequently we identified miR-146a-5p as the functional component of these exosomes. miR-146a-5p-modified exosomes exerted better effects than unmodified exosomes reducing the deleterious effects of neurotoxic astrocytes by inhibiting Traf6 and Irak1, mediators of signaling during the activation of the canonical NF-κB pathway, thereby promoting neurological function recovery after SCI.

## Materials and methods

### Ethics statement

Ethics approval for the experiments was obtained from the Animal Ethics Committee of South China Agricultural University (Ethics number: 2021d119). All experiments were performed following relevant laws and institutional guidelines of the Animal Ethics Committee of South China Agricultural University. This study did not involve human subjects.

### Animal experiment protocols

Female Sprague–Dawley rats were purchased from Hunan SJA Laboratory Animal Co., Ltd. (Hunan, China) and were housed in a specific pathogen-free facility with controlled temperature and humidity (temperature 22 ± 1℃; humidity, 65%-70%) with free access to food and water. Animal experiments were conducted according to protocols approved by the Animal Ethics Committee of South China Agricultural University (detailed animal information is provided in Additional file 1).

### Exosome purification and characterization

When Passage 5 hUCMSCs reached 80% confluency, the culture medium was replaced with exosome-depleted fetal bovine serum (FBS; Gibco, Australia), and the cells were incubated for an additional 48 h. The culture medium was collected, and exosomes were isolated from the supernatants of this medium with Total Exosome Extraction Reagent (Invitrogen, Carlsbad, California, USA) following the manufacturer’s instructions.

Exosomes isolated from supernatants were diluted in PBS (concentration 200 μg/100 μl) after we finished the quantification of protein concentration. The distribution of vesicle diameters was analyzed with a NanoSight NS300 system (Malvern Panalytical, Malvern, UK). Morphological analysis was carried out by transmission electron microscopy (TEM; Hitachi HT-7700, Tokyo, Japan). The exosome protein concentration was quantified using a bicinchoninic acid protein (BCA) assay kit (Thermo Fisher, Boston, MA, USA). Primary antibodies against CD9 (Abcam, Cambridge, UK), CD63 (Abcam, Cambridge, UK), and β-actin (CST, Boston, MA, USA) were used to identify the surface markers of exosomes by western blotting (detailed methods and materials are provided in Additional file 2, Additional file 3, Additional file 4 and Additional file 5).

### Statistical analyses

All data are presented as the mean ± standard deviation (SD). GraphPad Prism 9.0 software (San Diego, CA, USA) was used to calculate the *P* values and generate graphs. We performed Student’s t tests for two-group comparisons and one-way analyses of variance (one-way ANOVAs) for comparisons involving more than two-groups. A *P* value < 0.05 was considered statistically significant.

## Results

### Identification of hUCMSCs, primary astrocytes and hUCMSC-derived exosomes

hUCMSCs were provided by Guangzhou Saliai Stem Cell Science and Technology Co., Ltd. hUCMSCs from Passage 3 were identified on the basis of morphology and flow cytometry analyses. Morphologically, the cells at 80% confluency presented with a spindle-like shape. Flow cytometry was performed to identify surface makers, and the results confirmed that the stem cell markers CD73, CD90, and CD105 were highly expressed, while the nonstem cell markers CD34, CD45, and HLA-DR were not expressed (Additional file 6A). Primary astrocytes were characterized by immunostaining for GFAP, S100β, and Vimentin (Additional file 6B). We isolated exosomes from the supernatants of the hUCMSCs using Total Cell Exosome Extraction Kit. Nanoparticles isolated from cell supernatants presented as vesicles with a round-cup shape, as assessed via TEM (Additional file 6C). Nanoparticle tracking analysis (NTA) revealed that the size of the vesicles ranged from 30 to 150 nm. (Additional file 6D). The typical markers (CD9 and CD63) of the exosomes were also detected in these vesicles, which did not express β-actin (Additional file 6E). Taken together, the data indicated that the vesicles obtained were exosomes.

### Exosomes reduced the toxicity of neurotoxic astrocytes in vitro and in vivo

**Fig. 1:**
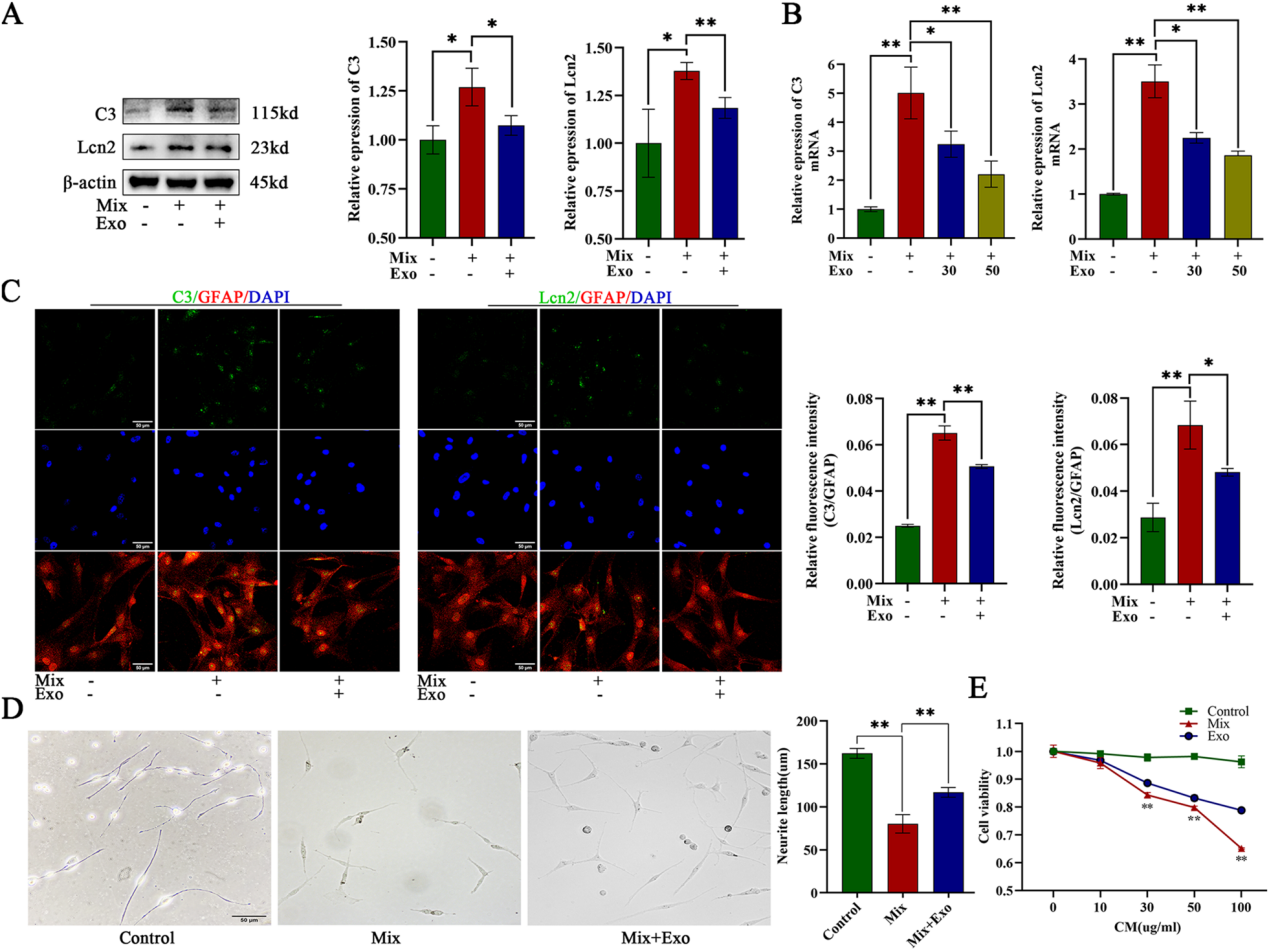
Exosomes reduced toxicity of neurotoxic astrocytes in vitro. **a,** **b** Representative western blot images and quantitative analysis of C3 and Lcn2 gene expression in control astrocytes, mixture (TNFα, IL1β, C1q)-induced astrocytes (neurotoxic astrocytes) and exosomes treated neurotoxic astrocytes. **c** Representative immunostaining images of GFAP (red)/C3 (green)/Lcn2 (green) and the quantification of the relative immunofluorescence intensity of C3 and Lcn2 in each group. Scale bar = 50 μm. **d** Representative images and quantitative analysis of neurite length in NGF-stimulated PC12 cells treated with conditioned medium (CM) of primary astrocytes (Control), neurotoxic astrocytes (Mix) and exosomes treated neurotoxic astrocytes (Mix-Exo). Scale bar = 50 μm. **e** Cell viability of PC12 cells treated with conditioned medium of different groups. Data above are represented as mean ± SD. *, *p* < 0.05; **, *p* < 0.01. C3, complement c3. Lcn2, lipocalin-2. NGF neuronal growth factor. TNFα, tumor necrosis factor α. IL1β, interleukin 1β. C1q, complement 1q

We first investigated whether the exosomes derived from the hUCMSCs can reduce the toxicity of neurotoxic astrocytes. We generated astrocytes with a neurotoxic phenotype by treating primary astrocytes with a mixture of IL-1α (3 ng/mL), TNF-α (30 ng/mL), and C1q (400 ng/mL) for 24 h. The visualization of PKH26 labeled exosomes via confocal microscopy confirmed the successful endocytosis of exosomes by neurotoxic astrocytes (Additional file 6F). After administration of the induction mixture, the expression of markers (complement C3, lipocalin-2 (Lcn2)) of the neurotoxic astrocytes was significantly elevated at the protein and mRNA levels, which was subsequently partially reduced by exosome treatment (Fig. 1a, b, c).To investigate whether exosomes reduce the toxic effect of neurotoxic astrocytes on neurons, we pretreated PC12 cells with NGF to induce the differentiation and growth of neurites and then cultured these cells with conditioned medium (CM) obtained from neurotoxic astrocytes culture (CM-Mix) or CM from culture of neurotoxic astrocytes treated with exosomes (CM-Mix + Exo). The results revealed that the CM-Mix caused a significant increase in neurite decline and the length of the neurites in this culture was extremely short (Fig. 1d). When cultured with CM-Mix + Exo, more neurites of PC12 cells were preserved (Fig. 1d). The results also revealed that CM from neurotoxic astrocyte culture significantly reduced the viability of PC12 cells in a dose dependent manner, while CM-Mix + Exo have weaker influence on PC12 cells viability (Fig. 1e). These results demonstrated that the CM of neurotoxic astrocyte exerted powerful toxic effect that hindered the growth and neurite extension of PC12 cells, and these adverse effects were partially reversed by exosomes treatment.

**Fig. 2:**
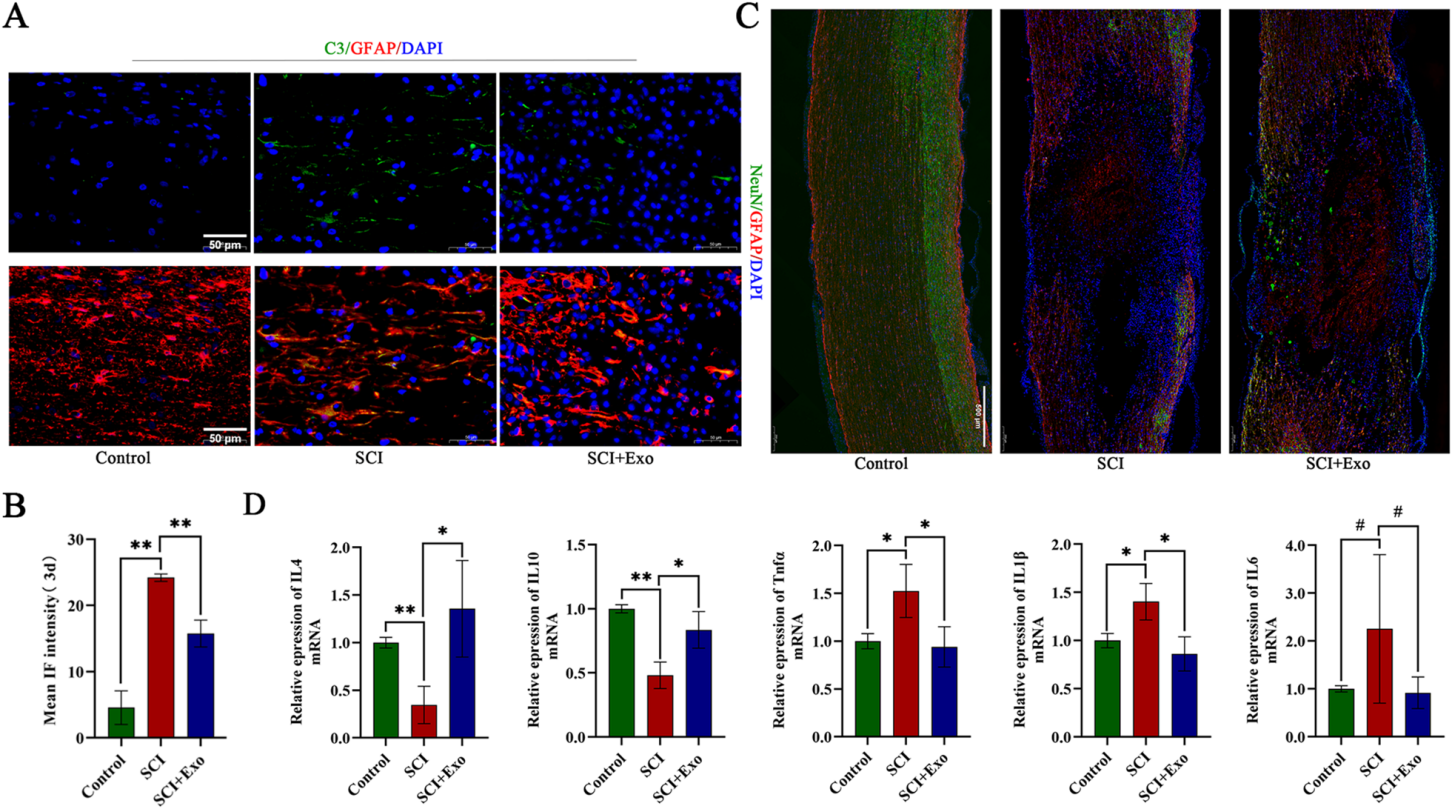
Exosomes reduced toxicity of neurotoxic astrocytes in vivo. **a**, **b** Representative immunostaining images of C3 (green)/GFAP (red) in sham-operated rats (the Control group), SCI rats (the SCI group), SCI rats treated with Exo (the SCI + Exo group) and the quantification of the relative immunofluorescence intensity of C3 in each group at 3dpi (detailed regions where the representative image selected from are provided in Additional file 9). Scale bar = 50 μm. **c** Representative immunofluorescence images of Neu-N (green)/GFAP (red) at 3 dpi in each group. Scale bar = 500 μm. **d** Quantitative analysis of mRNA expression of anti-inflammation cytokines (IL4, IL10) and pro-inflammation cytokines (TNFα, IL1β, IL6) in spinal cord tissue of each group. Data above are represented as mean ± SD. *, *p* < 0.05; **, *p* < 0.01; #, *p* > 0.05. IL4 interleukin 4, IL6 interleukin 6, IL10 interleukin 10, dpi days post-injury

To further investigate the effect of exosomes in vivo, exosomes were injected intravenously into rats with SCI. Tracking of PKH-26-labeled exosomes by immunofluorescence confirmed the successful delivery of the exosomes to lesions (Additional file 7A). Three days post-injury (dpi), immunofluorescence showed that C3-positive astrocytes were abundant around the spinal cord lesions, and the number of neurons stained by Neu-N was obviously decreased (Fig. 2a–c). These results demonstrated that neurotoxic astrocytes were activated, and correspondingly, neural tissue was severely damaged in the SCI group. Fortunately, the group with administered exosomes showed a decrease in the number of neurotoxic astrocytes and a higher degree of neural tissue preservation 3 dpi (Fig. 2a–c). We analyzed the expression of proinflammatory factors (TNFα, IL-1β, and IL-6) and anti-inflammatory factors (IL-4 and IL-10). The results showed that the expression of the proinflammatory factors was significantly elevated at the mRNA level, while that of the anti-inflammatory factors was much lower in the SCI group (Fig. 2d). In contrast, exosome administration reversed the increase in proinflammatory mRNA expression and promoted the expression of anti-inflammatory factors (Fig. 2d). These results indicate that MSC-Exos reduced the toxicity of neurotoxic astrocytes and increased neural preservation in rats with SCI. Inflammation was also relieved by the administration of exosomes. Taken together, these results indicated that exosomes derived from hUCMSCs reduced the toxicity of neurotoxic astrocytes to preserve neurons in vitro and in vivo.

### Exosomal miR-146a-5p was critical for reducing the toxicity of neurotoxic astrocytes

**Fig. 3:**
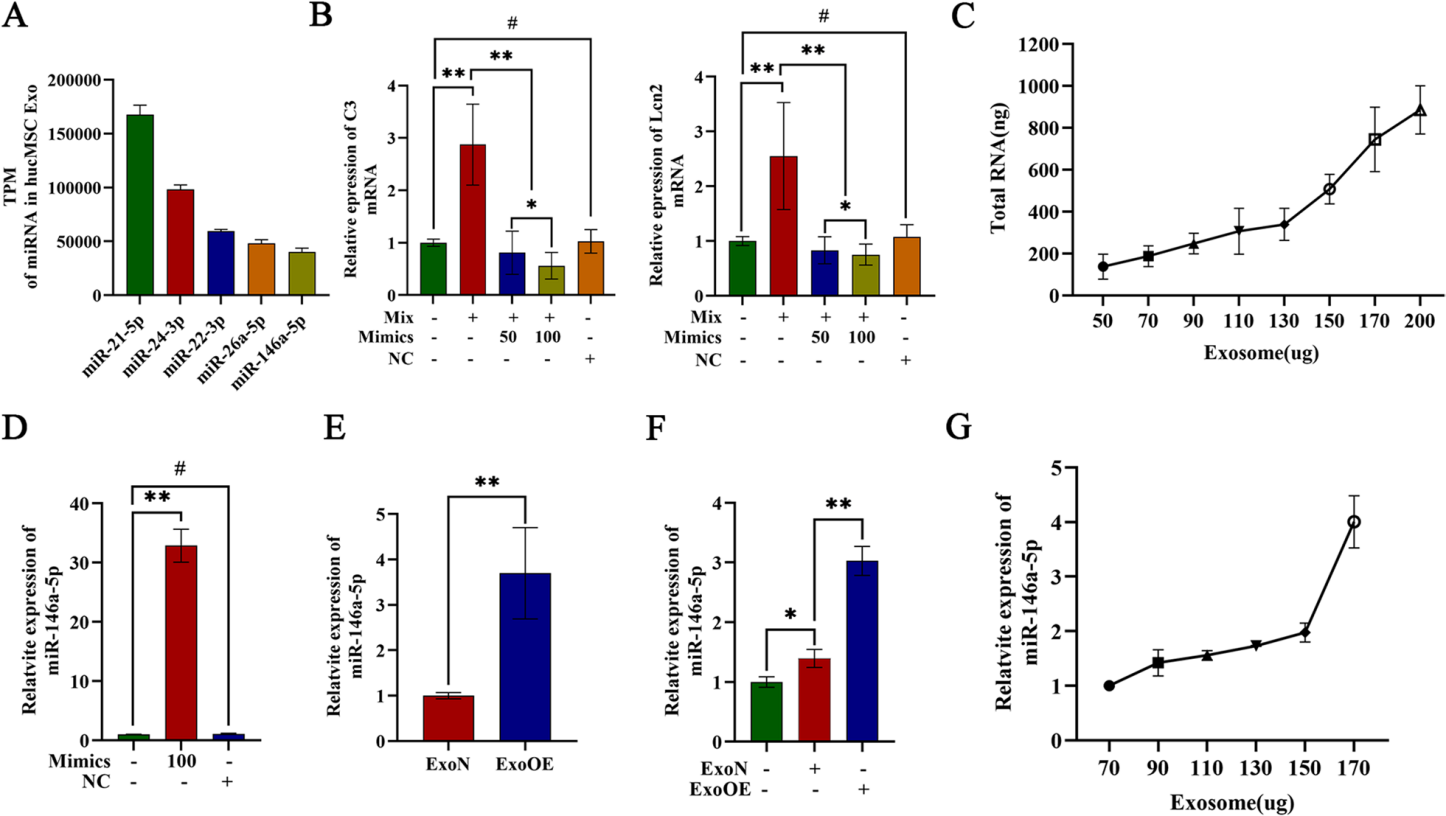
Exosomal miR-146a-5p was critical for reducing the toxicity of neurotoxic astrocytes. **a** The 5 most abundant miRNAs expressed in hUCMSCs-Exo. **b** Quantitative analysis of mRNA expression of C3 and Lcn2 in astrocytes, neurotoxic astrocytes and neurotoxic astrocytes treated with 50/100 nM mimics of miR-146a-5p or negative control mimics (NC). **c** The linear relationship between total RNAs content and exosomal proteins content. **d** Quantitative analysis of miR-146a-5p expression in hUCMSCs, miR-146a-5p or mimics NC over-expressed hUCMSCs. **e** Quantitative analysis of miR-146a-5p expression in normal exosomes (ExoN) and miR-146a-5p-modified hUCMSCs-derived exosomes (ExoOE). **f** Quantitative analysis of miR-146a-5p expression of astrocytes, ExoN treated astrocytes and ExoOE treated astrocytes. **g** The linear relationship between miR-146a-5p expression and exosomal proteins content. Data above are represented as mean ± SD. *, *p* < 0.05; **, *p* < 0.01; #, *p* > 0.05

miRNAs are the most abundant nucleic acids in MSC-Exos and are the main functional components of these exosomes. Exosomes play critical roles in many diseases like inflammation, trauma, tumor et al. To better understand the exosomal miRNA profile and underlying functions, in a previous study, we preformed absolute quantitative sequencing, and the results were deposited in the NCBI GEO database (GSE 159814). Approximately 990 miRNAs were identified in MSC-Exos, and the 5 most abundant miRNAs were miR-21-5p, miR-24-3p, miR-22-3p, miR-26a-5p, and miR-146a-5p (Fig. 3a). To investigate the function of the miRNAs detected in these exosomes on neurotoxic astrocytes, we pretransfected primary astrocyte with miRNA mimics followed by an induction mixture. The results of RT-qPCR assays revealed that miR-146a-5p, which was abundant in MSC-Exos, significantly suppressed mixture-induced expression of neurotoxic astrocyte markers (C3 and Lcn2) in dose depend manner (Fig. 3b). The results of the other 4 abundant miRNAs did not show significant effects (Additional file 8). Considering these results and the therapeutic effects of exosomes, we presumed that miR-146a-5p-modified (overexpressed) hUCMSCs-Exos (ExoOEs) exerted more powerful function than normal hUCMSCs-Exos (ExoNs) in reducing the toxicity of neurotoxic astrocytes. To investigate the function of miR-146a-5p-modified MSC-Exos, we first determined the level of total RNAs and miR-146a-5p in the exosomes. A concentration analysis showed that the total RNA content was positively correlated with the concentration of exosomal proteins, and the expression of miR-146a-5p showed the same relationship as that found for exosomal proteins (Fig. 3c, g). Then, we constructed miR-146a-5p-overexpressing hUCMSCs by transfecting miR-146a-5p mimics using Lipofectamine. The results showed the efficiency of the transfection procedure (Fig. 3d). Exosomes were isolated from supernatants of normal hUCMSCs culture or miR-146a-5p-overexpressed hUCMSCs culture. RT-qPCR revealed a significant increased miR-146a-5p level in the ExoOEs than that in ExoNs (Fig. 3e). In astrocytes treated with ExoNs, the expression level of miR-146a-5p was increased, and this level was significantly higher in astrocytes treated with ExoOEs (Fig. 3f).

### miR-146a-5p-modified exosomes reduced the toxicity of neurotoxic astrocytes in vitro

**Fig. 4:**
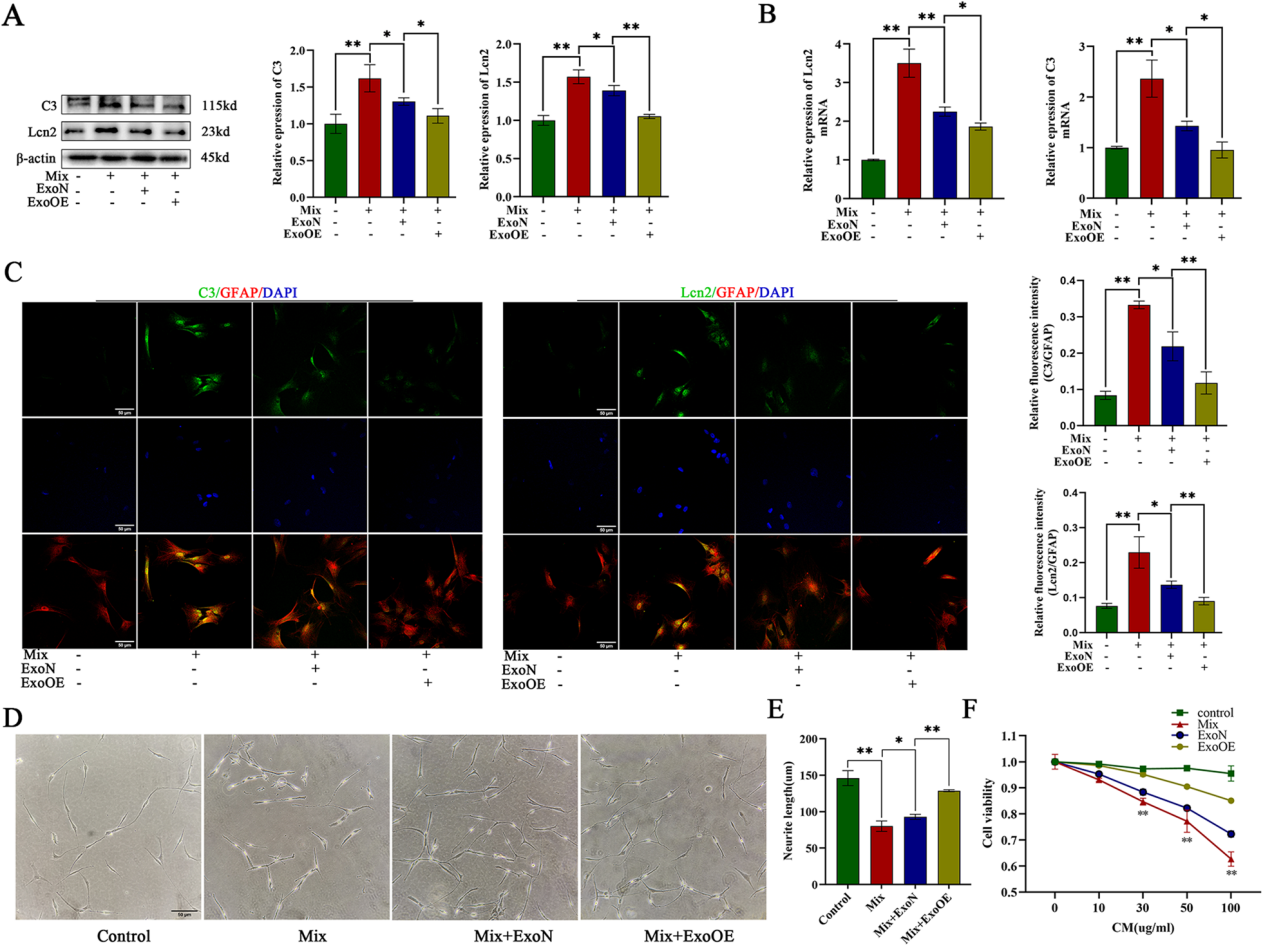
miR-146a-5p-modified exosomes reduced toxicity of neurotoxic astrocytes in vitro. **a b** Representative western blot images and quantitative analysis of C3 and Lcn2 gene expression in control astrocytes, mixture(TNFα, IL1β, C1q)-induced astrocytes (neurotoxic astrocytes) and ExoN/ExoOE treated neurotoxic astrocytes. **c** Representative immunostaining images of GFAP (red)/C3 (green)/Lcn2 (green) and the quantification of the relative immunofluorescence intensity of C3 and Lcn2 in each group. Scale bar = 50 μm. **d,** **e **Representative images and quantitative analysis of neurite length in NGF-stimulated PC12 cells treated with conditioned medium of primary astrocytes (Control), neurotoxic astrocytes (Mix) and ExoN/ExoOE treated neurotoxic astrocytes (Mix-ExoN or Mix-ExoOE). Scale bar = 50 μm. **f** Cell viability of PC12 cells treated with conditioned medium of different groups. Data above are represented as mean ± SD. *, *p* < 0.05; **, *p* < 0.01. ExoN normal exosome, ExoOE miR-146a-5p-modified exosome

To determine whether ExoOEs exert a more powerful reducing effect on the toxicity of neurotoxic astrocytes than ExoNs, we induced neurotoxic phenotype acquisition by astrocytes by treating primary astrocytes with induction mixture and treated neurotoxic astrocytes with ExoNs or ExoOEs. The RT-qPCR results revealed that both ExoNs and ExoOEs reduced the mRNA expression of markers C3 and Lcn2 in the neurotoxic astrocytes, and that the ExoOEs exerted a better effect than the ExoNs (Fig. 4b). These functions were confirmed at the protein level by the western blot and immunofluorescence results (Fig. 4a, c). To further investigate whether ExoOEs lead to greater neurite preservation, we cocultured PC12 cells with CM-Mix, CM-Mix + ExoN or CM-Mix + ExoOE after induction by NGF. Analysis of neurite length in the different groups revealed that CM-Mix + ExoN and CM-Mix + ExoOE both preserved PC12 cells neurite growth to different degrees (Fig. 4d, e). After coculture with CM-Mix + ExoOE, the PC12 cells showed greater preservation of neurite length (Fig. 4d, e). The results of cell count kit-8 (CCK-8) assay also revealed that the CM of exosome-treated neurotoxic astrocytes showed less toxic effect on PC12 cells, while CM-Mix + ExoOE had an even smaller toxic effect (Fig. 4f). Taken together, these results demonstrated that miR-146a-5p-modified exosomes exerted powerful effects by reducing toxicity of neurotoxic astrocytes.

### miR-146a-5p-modified exosomes reduced the toxicity of neurotoxic astrocytes in vivo and promoted the functional recovery of rats with SCI

**Fig. 5:**
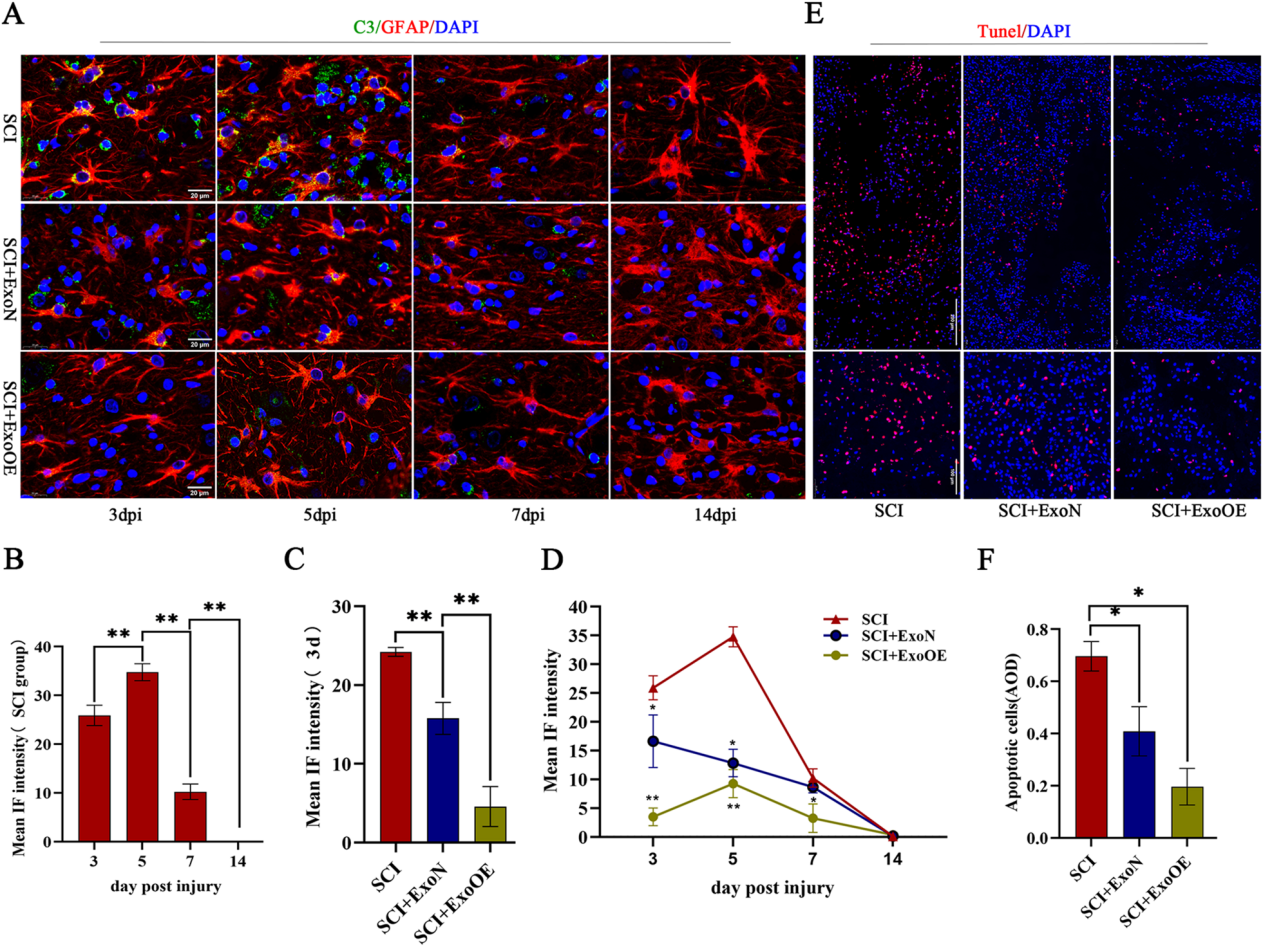
miR-146a-5p-modified exosomes reduced toxicity of neurotoxic astrocytes in vivo. **a** Representative immunostaining images of C3(green)/GFAP (red) in rats of SCI (the SCI group), SCI rats treated with ExoN and ExoOE (the SCI + ExoN group and the SCI + ExoOE group) 3dpi, 5dpi, 7dpi and 14 dpi (detailed regions where the representative image selected from are provided in Additional file 10) Scale bar = 20 μm. **b** Quantification of the mean immunofluorescence intensity of C3 in rats with SCI at 3rd, 5th, 7th and 14th dpi. **c, d** Mean immunofluorescence intensity of C3 in rats of three groups at 3dpi 5dpi, 7dpi and 14 dpi. **e, f** Representative images and quantitative analysis of TUNEL staining in rats of SCI, SCI + ExoN and SCI + ExoOE group at 3dpi. Scale bar = 100 μm. Data above are represented as mean ± SD. *, *p* < 0.05; **, *p* < 0.01. #, *p* > 0.05

To determine whether ExoOEs exert therapeutic effects similar to those observed in vitro, we constructed SCI model rats and treated them with PBS (the SCI group), ExoN (the SCI + ExoN group), ExoOE (the SCI + ExoOE group). First, we observed the natural development of C3-positive astrocytes at different time points (3 dpi, 5 dpi, 7 dpi, and 14 dpi) through immunofluorescence. The results showed that the number of C3-positive astrocytes increased significantly 3 dpi and reached a peak 5 dpi. However, it decreased dramatically 7 dpi and had almost disappeared by 14 dpi (Fig. 5a, b). After administration of ExoNs or ExoOEs, the number of C3-positive astrocytes in both groups was decreased significantly, and ExoOEs showed an even stronger ability to reduce C3-positive astrocytes at 3 dpi, 5 dpi, and 7 dpi, although there were no differences between the SCI and SCI + ExoN groups 7 dpi (Fig. 5a, c, d). Interestingly, by 14 dpi, C3-positive astrocytes were undetectable in all three groups (Fig. 5a, d). Astrocytes adjacent to lesions in the three groups 14 dpi were larger and exhibited elongated morphologies with overlapping cell processes. TUNEL staining revealed a large number of apoptotic cells around the spinal cord lesion in the SCI group 3 dpi and this number was reduced by the administration of ExoNs in SCI + ExoN group. The SCI + ExoOE group showed fewer apoptotic cells than the other groups (Fig. 5e, f).

**Fig. 6:**
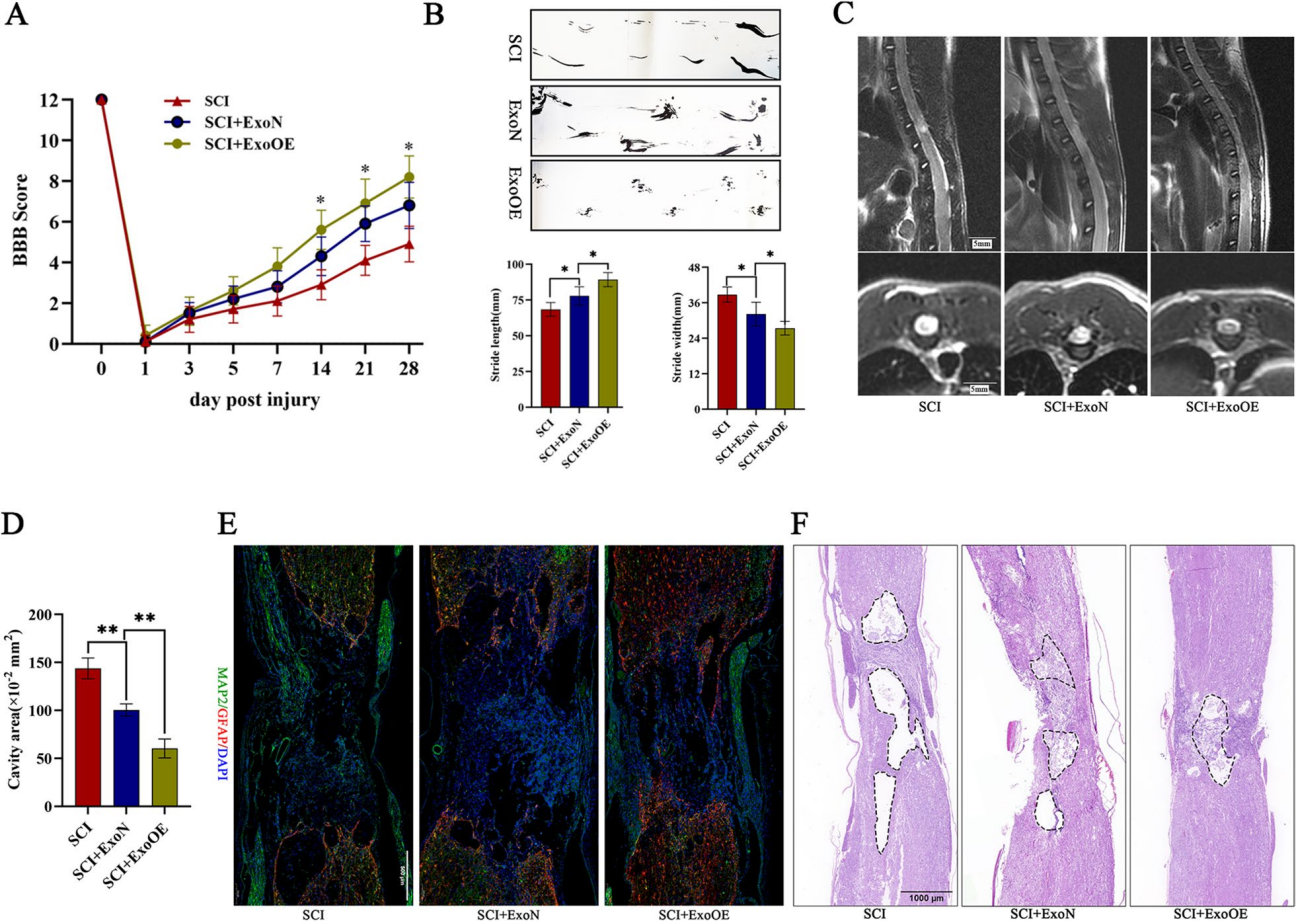
miR-146a-5p-modified exosomes promoted the functional recovery of rats with SCI. **a** BBB scores of rats in three groups on the 1st, 3rd, 5th, 7th, 14th, 21st, 28th dpi (n = 10/group). **b** Representative footprints of rats walking in 28th dpi in each group and quantification of the footprints analysis. **c, d** Quantitative analysis and representative MRI images of the lesion area of rats in each group at 28th dpi. Scale bar = 5 mm **e** Representative immunostaining images of MAP2 (green) /GFAP (red) in spinal cord tissue of each group at 28th dpi. Scale bar = 500 μm. **f** Representative images of the lesion size (HE staining) in the three groups of rats at 28th dpi. Scale bar = 1000 μm Data above are represented as mean ± SD. *, *p* < 0.05; **, *p* < 0.01

To further investigate the therapeutic effects of ExoNs and ExoOEs in vivo, we carried out a series of experiments to evaluate the functional recovery of SCI rats in the different groups. The successful delivery of miR-146a-5p was confirmed by RT-qPCR (Additional file 7B). The Basso, Beattie and Bresnahan (BBB) scale used to evaluate locomotor function of hindlimbs showed that exosomes improved functional recovery, which had been compromised by SCI. ExoOEs performed better than ExoNs in our experiment (Fig. 6a). The footprint analysis of the three groups confirmed the BBB scale results (Fig. 6b). The MRI scans used to detect lesions in vivo indicated that exosomes decreased the lesion area, in contrast to the area in the SCI group, and ExoOEs exhibited a greater ability to reduce lesion volume (Fig. 6c, d). We also applied immunofluorescence to investigate the function of exosomes in neurons, and neuronal marker (MAP2) staining revealed that ExoOEs preserved more neural tissue than ExoNs, but both showed the therapeutic effects in terms of neuron preservation (Fig. 6e). Hematoxylin–eosin (HE) staining indicated that the ExoOE group had smaller lesions than the ExoN group, but both groups showed reduced lesion volume (Fig. 6f).

These results demonstrated that ExoOEs were more powerful than ExoNs, and that miR-146a-5p strengthened the capability of exosomes to reduce toxicity of neurotoxic astrocytes and markedly promoted the functional recovery of SCI in rats.

### Exosomal miR-146a-5p regulates Irak1 and Traf6 expression by targeting their mRNA 3’-UTRs

**Fig. 7:**
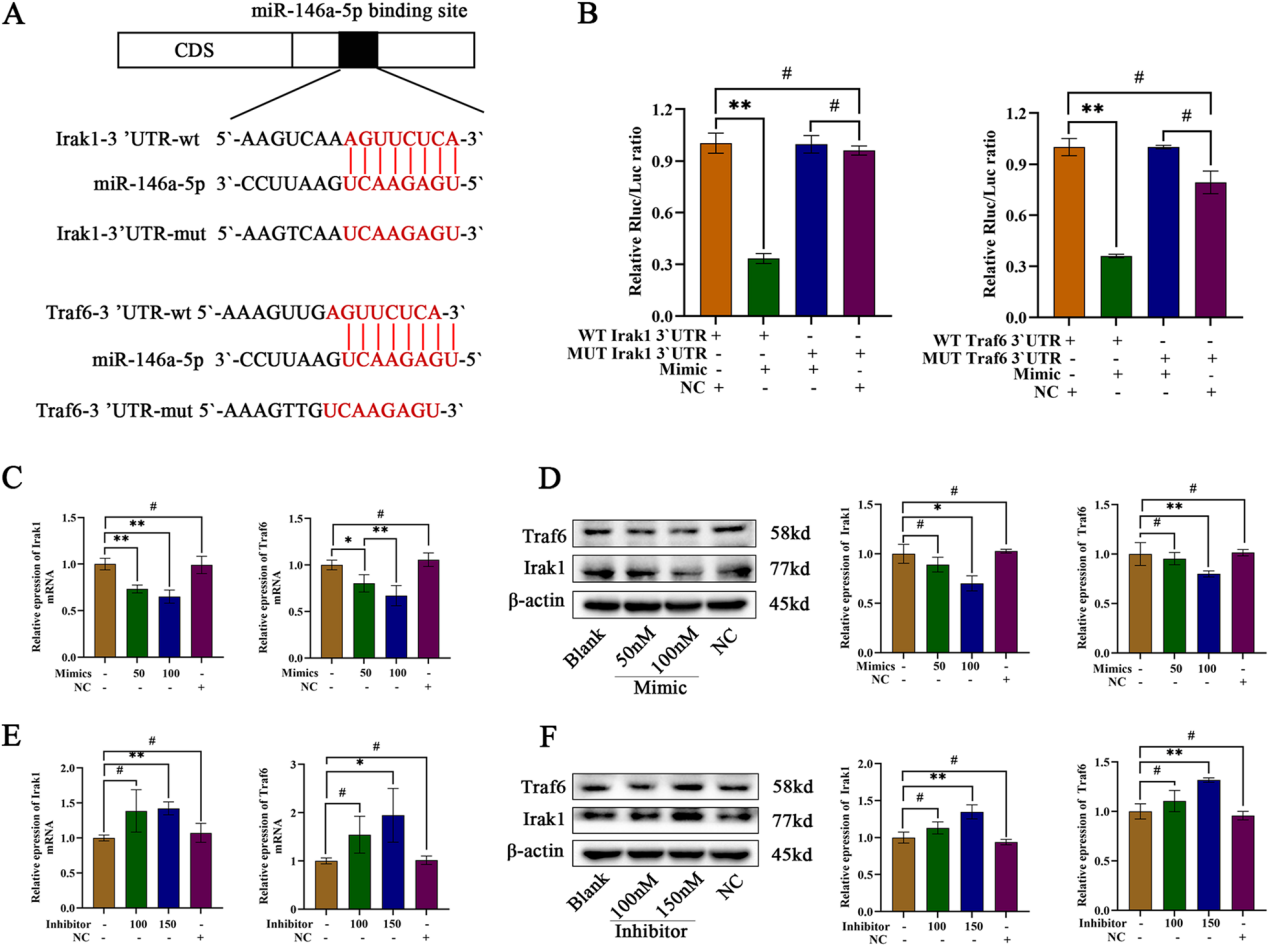
Exosomal miR-146a-5p regulates Irak1 and Traf6 by targeting their mRNA 3’-UTR. **a** Predicted binding sites of miR-146a-5p on Irak1 and Traf6 mRNAs. **b** Luciferase reporter assay of predicted binding sites of miR-146a-5p in HEK293 cells. **c d** Irak1 and Traf6 gene expressions in astrocytes transfected with different doses of miR-146a-5p mimics and (**e f**) inhibitors assayed by western blot and QRT-PCR. Data above are represented as mean ± SD. *, *p* < 0.05; **, *p* < 0.01. #, *p* > 0.05. WT wild type, MUT mutant, QRT-PCR quantitative real-time polymerase chain reaction, Traf6 TNF receptor associated factor 6, Irak1 interleukin 1 receptor associated kinase 1

To further investigate the underlying mechanism of exosomal miR-146a-5p in the ExoOE regulation of neurotoxic astrocytes, we focused on the downstream targets of miR-146a-5p. According to the online database used for miRNA target prediction, we focused on TNF receptor associated factor 6 (TRAF6) and interleukin 1 receptor associated kinase 1 (IRAK1) and hypothesized that they were direct targets of miR-146a-5p (Fig. 7a). To verify whether miR-146a-5p targets the respective 3`-UTR of TRAF6 and IRAK1, we constructed wild-type (WT) and mutant (MUT) mRNA 3`-UTR sequences in TRAF6 and IRAK1 based on binding-site prediction. We cloned these genes into the psiCHECK-2 dual luciferase miRNA target expression vector. Plasmids were cotransfected with miRNA mimics into HEK293 cells. Relative luciferase activity was significantly decreased when the mimics were cotransfected with the WT-3`-UTR of TRAF6 or IRAK1 mRNA, but changes in luciferase activity was not be observed when mimics were cotransfected with the MUT-3`-UTR of TRAF6 or IRAK1 mRNA (Fig. 7b). Marked alterations in luciferase activity were not be observed when a scramble sequence (negative control (NC) group) were cotransfected with the WT-3`-UTR or MUT-3`-UTR of target gene mRNAs (Fig. 7b). The scramble control did not influence luciferase activity (Fig. 7b).

Furthermore, we investigated whether miR-146a-5p regulates TRAF6 and IRAK1 gene expression at the transcriptional or posttranscriptional level. First, we overexpressed miR-146a-5p by transfecting mimics into astrocytes, and we found that the expression of the TRAF6 and IRAK1 genes was decreased at both the mRNA and protein levels as the doses were increased (Fig. 7c, d). Then we transfected inhibitors into the astrocytes to knockdown miR-146a-5p expression, and the mRNA and protein levels of TRAF6 and IRAK1 were found to be increased as the doses were increased (Fig. 7e, f). These results suggested that the TRAF6 and IRAK1 genes are downstream targets of miR-146a-5p.

### miR-146a-5p-modified exosomes reduced the toxicity of neurotoxic astrocytes by targeting Irak1 and Traf6

**Fig. 8:**
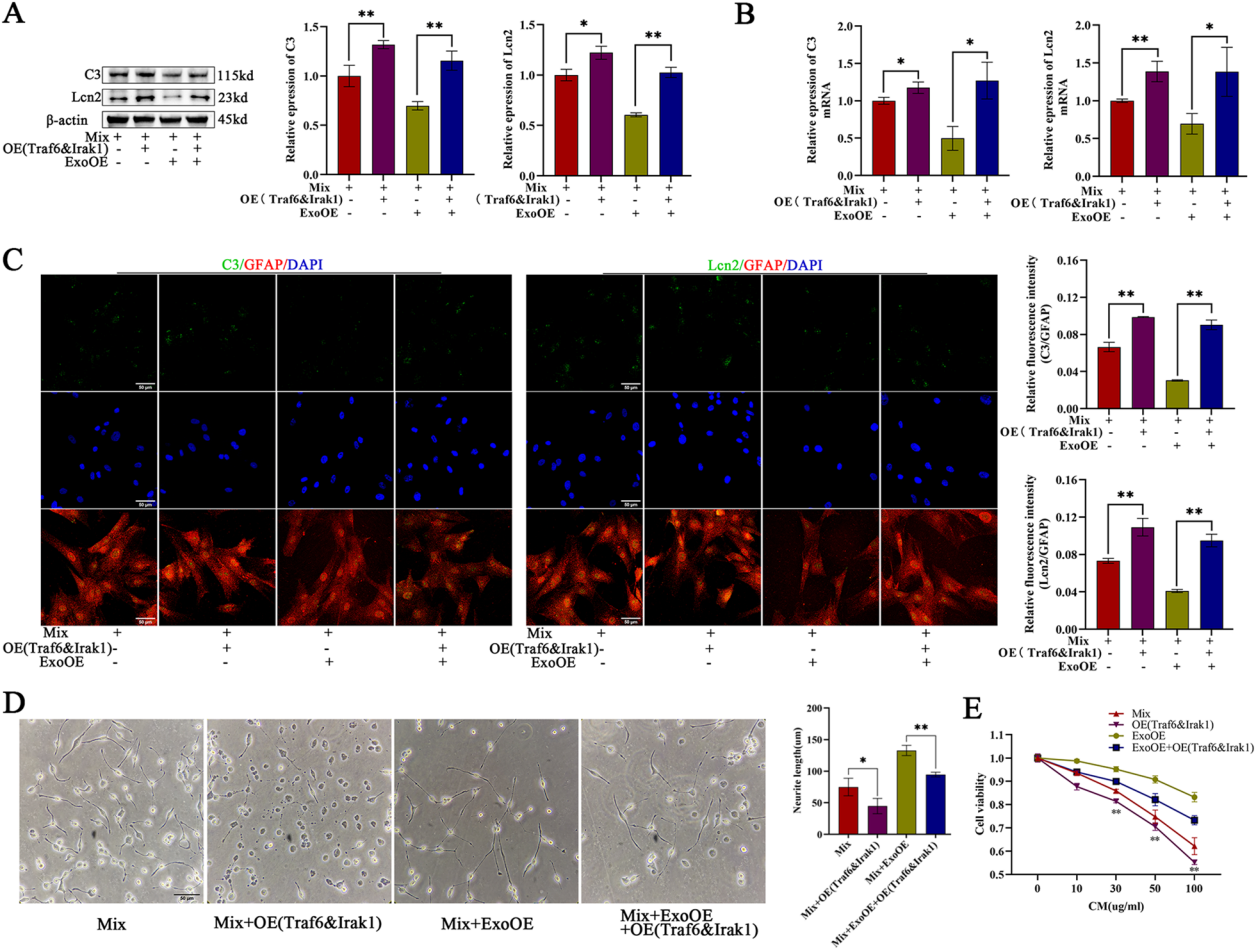
miR-146a-5p-modified exosomes reduced the toxicity of neurotoxic astrocytes by targeting Irak1 and Traf6. **a, b** Representative western blot images and quantitative analysis of C3 and Lcn2 gene expression in mixture(TNFα, IL1β, C1q)-induced astrocytes (neurotoxic astrocytes), Irak1&Traf6 over-expressed neurotoxic astrocytes, ExoOE treated neurotoxic astrocytes and ExoOE treated neurotoxic astrocytes followed by over-expressing Irak1&Traf6. **c** Representative immunostaining images of GFAP (red)/C3 (green)/Lcn2 (green) and the quantification of the relative immunofluorescence intensity of C3 and Lcn2 in each group. Scale bar = 50 μm. **d** Representative images and quantitative analysis of neurite length in NGF-stimulated PC12 cells treated with conditioned medium of neurotoxic astrocytes (Mix), Irak1&Traf6 over-expressed neurotoxic astrocytes (OE(Traf6&Irak1)), ExoOE treated neurotoxic astrocytes(ExoOE) and ExoOE treated neurotoxic astrocytes followed by over-expressing Irak1&Traf6(ExoOE + OE(Traf6&Irak1)).Scale bar = 50 μm. **e** Cell viability of PC12 cells treated with conditioned medium of different groups. Data above are represented as mean ± SD. *, *p* < 0.05; **, *p* < 0.01

To further explore the relationship between miR-146a-5p-modified exosomes and Irak1/Traf6 in the regulation of neurotoxic astrocytes toxicity, a series of gain- and loss-of-function experiments were carried out. Since the marker expression of neurotoxic astrocytes was extremely low in primary astrocytes, we performed these gain- or loss-of-function experiments on neurotoxic astrocytes. Overexpression of the targets genes (Irak1/Traf6) resulted in higher expression of neurotoxic astrocyte markers, while administration of ExoOEs showed the opposite results at the mRNA and protein levels (Fig. 8a-c). Then, we overexpressed the target genes (Irak1/Traf6) on the basis of ExoOE administration to investigate whether they neutralize the knockdown effects of the ExoOEs. The results showed that the effects of the ExoOEs were largely reversed by overexpression of the target genes (Fig. 8a-c). These results were also confirmed by the alteration of the neurotoxic phenotype of astrocytes, as determined by neurite length and a cell viability analysis in coculture with PC12 cells and different conditioned medium (Fig. 8d, e). Overexpression of the target genes (Irak1/Traf6) reversed the ExoOE effects on neurite length and PC12 cells preservation (Fig. 8d, e). Taken together, these results demonstrated that miR-146a-5p-modified-exosomes reduced the toxicity of neurotoxic astrocytes by targeting Irak1 and Traf6.

### miR-146a-5p-modified exosomes reduced the toxicity of neurotoxic astrocytes by targeting the Traf 6/Irak1/NFκB pathway

**Fig. 9:**
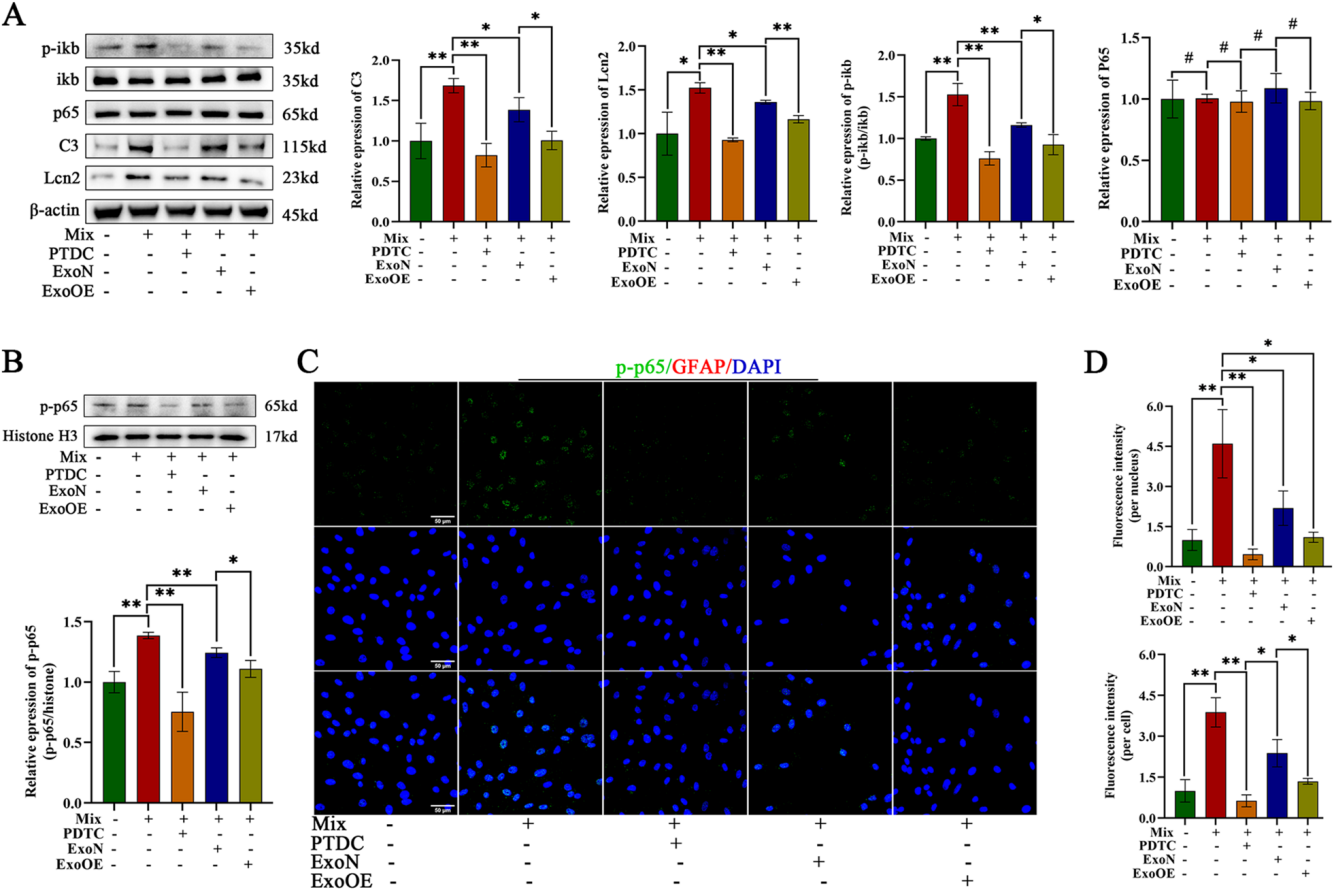
miR-146a-5p-modified exosomes reduced toxicity of neurotoxic astrocytes by targeting Traf 6/Irak1/NFκB pathway. **a** Representative images and quantification of western blots for C3, Lcn2 and critical mediators of NFκB signaling pathway in control astrocytes, neurotoxic astrocytes (Mix), ExoN/ExoOE treated neurotoxic astrocytes and PDTC treated neurotoxic astrocytes. **b** Representative images and quantification of western blots of p-p65 in nucleus of astrocytes with different administrations. **c, d** Immunostaining images of p-p65 in nucleus and quantification of the mean immunofluorescence intensity of total p-p65 per cell and nuclear p-p65 per cell. Scale bar = 50 μm. Data above are represented as mean ± SD. *, *p* < 0.05; **, *p* < 0.01; #, *p* > 0.05. PDTC ammonium pyrrolidine dithiocarbamate, P-p65 phospho-NFκB p65, P-ikb phospho-ikb

Reports in the literature have suggested that the activation of neurotoxic astrocytes is mediated by the NFκB signaling pathway. Traf 6 and Irak1 are mediators of NFκB signaling. Our western blot results revealed that the induction mixture increased the relative expression of phosphorylated (p)-ikb and p-p65 without altering the total expression of ikb or p65 (Fig. 9a). These effects were partially blocked by the administration of ExoNs and mostly blocked by the administration of ExoOEs (Fig. 9a). Interestingly, ExoOEs-induced effects similar to those of ammonium pyrrolidine dithiocarbamate (PDTC), which is an inhibitor of the NFκB signaling pathway (Fig. 9a). The protein expression of p-p65 in the nucleus was also increased in cells exposed to the induction mixture, but it was reversed by ExoOE treatment, similar to the effect observed after the administration of PDTC (Fig. 9b). These results were verified by immunofluorescence assays (Fig. 9c d). Then, we focused on the markers C3 and Lcn2 of neurotoxic astrocytes. The results showed that when we blocked the activation of NFκB signaling with PDTC, the protein expression of C3 and Lcn2 was markedly decreased (Fig. 9a). Taken together, these results demonstrated that miR-146a-5p-modified exosomes reduced the toxicity of neurotoxic astrocytes by targeting the Traf 6/Irak1/NFκB pathway.

## Discussion

SCI, as a central nervous system insult, triggers multiple responses involving neurons, glia and nonneural cells. Robust activation of microglia and inflammatory responses subsequently induce astrocyte reactivity, causing neurotoxic phenotype changes and exerting powerful neural toxicity [9]. Previous studies have indicated that exosomes share therapeutic effects similar to those of MSCs in inhibiting inflammation and converting the microglial phenotype to the M2 phenotype [11, 12]. We induced the acquisition of the neurotoxic phenotype in astrocytes by applying a mixture of TNFα, IL-1α, and C1q as previously described [9], and the results demonstrated that the administration of exosomes reduced the neurotoxic effects of neurotoxic astrocytes to preserve PC12 cells and neurites. An in vivo study confirmed these results, with fewer number of neurotoxic astrocytes and greater neural tissue preservation. Our previous study had also shown that exosomes from hUCMSCs promoted neurite outgrowth [17]. Taken together, the data indicated that exosomes promoted neurological function recovery through multiple mechanisms. Neurotoxic astrocytes are critical targets through which exosomes exert their therapeutic effects on SCI.

miRNAs are the most abundant nucleic acids in exosomes and are among the main functional components in exosomes [18]. We previously quantified miRNA expression profiles using absolute quantitative sequencing [17]. miR-146a-5p, as one of the five most abundant cargo miRNAs in hUCMSC-exosomes, significantly reduced the expression of neurotoxic astrocyte markers in our study. These results indicated that miR-146a-5p was the critical functional component of these exosomes. miR-146a-5p has been previously shown to exert modulatory effects on inflammation and immune responses [19, 20]. However, oligonucleotides are susceptible to degradation, rapid clearance, and immune responses, which limit their efficiency [21]. Additionally, some factors, such as relatively low yield of exosomes from cell culture supernatant, limit exosomal functions [13]. Interestingly, exosomes possess advantages such as the capability to carry and protect nucleic acids, the capability to cross biological barriers, and the ability to target cells qualifying them as excellent drug delivery systems [14]. Lou et al. showed that miR-199a-modified exosomes from adipose tissue-derived MSCs improve hepatocellular carcinoma chemosensitivity [22].

To combine the respective advantages of miR-146a-5p and exosomes, we constructed miR-146a-5p-modified exosomes by overexpressing miRNAs in normal exosomes. The results showed that miR-146a-5p-modified exosomes performed better than normal exosomes in reducing neurotoxic astrocytes and restoring neurological function in SCI rats. In turn, these results confirmed the crucial role of miR-146a-5p and the superior therapeutic effects of modified exosomes.

The dynamics of neurotoxic astrocytes in acute SCI are of great significance to understand the pathology and management of the disease. Qian et al. showed that few neurotoxic astrocytes were apparent in the early stage of injury and that the number was increased significantly by 28 dpi [23]. Activation of microglia, inflammatory responses and trauma are considered as critical factors contributing to the reaction of neurotoxic astrocytes. Liddelow et al. [9] suggested that neurotoxic astrocytes form rapidly after CNS injury and are present in many human neurodegenerative diseases, which was consistent with our results. Our study suggested that the number of neurotoxic astrocytes increased over time, reaching a peak 5 dpi, and then returned to the normal level 14 dpi. From the perspective of treatment timing, the administration of exosomes in early stage of SCI is the optimal strategy to obtain the best therapeutic effects.

From an online database (miRDB.org) of predicted miRNA targets, we identified Traf6 and Irak1 as targets of exosomal miR-146a-5p, and our luciferase reporter and western blot analyses confirmed that they are miR-146a-5p targets. The results of a series of gain- and loss-of-function experiments confirmed that miR-146a-5p-modified exosomes reduced the neurotoxicity of neurotoxic astrocytes by inhibiting Traf6 and Irak1 expression.

Traf6 and Irak1 are signal mediators of canonical NFκB pathway activation. Previous studies have indicated that NFκB signaling is involved in inducing neurotoxic astrocytes in optic nerve crush and Alzheimer’s disease [9, 24]. In the present study, administration of an induction mixture activated NFκB signaling and induced the neurotoxic effect of astrocytes. Blocking the activation of the NFκB pathway by PDTC led to the opposite results which were consistent with the results of miR-146a-5p-modified exosomes experiments. These results indicated that miR-146a-5p-modified exosomes reversed the neurotoxic phenotype of astrocytes by inhibiting NFκB signaling cascades, and this mechanism was consistent with the negative feedback regulating role previously shown to be played by miR-146a-5p in activating the canonical NFκB pathway [25].

Although our results implied neurological improvement with the administration of miR-146a-5p-modified exosomes, which attenuated the toxic effects of neurotoxic astrocytes, problems and shortcomings remain. We cannot rule out the possibility that other exosomal miRNAs may have exhibited the same therapeutic effects as miR-146a-5p. Although modified and unmodified exosomes are regarded as promising therapeutics, considerable effort must be directed to address safety issues, such as immunogenic or toxic effects, before exosomes can be used in clinical applications.

## Conclusions

In summary, miR-146a-5p-modified exosomes exerted a more powerful effect than unmodified exosomes to promote the neurological function recovery of SCI in rats by dramatically reducing the toxic effects of neurotoxic astrocytes. These effects were mainly obtained by inhibiting the expression of Traf6 and Irak1, which are mediators of signaling during canonical NFκB pathway activation. To obtain the best therapeutic outcomes, exosomes should be administered within 7 dpi to attenuate astrocyte neurotoxic effects.

## Supplementary Information

**Additional file 1.** Animal protocol.

**Additional file 2.** Supplementary methods and materials.

**Additional file 3.** Primers of target genes.

**Additional file 4.** miRNA sequences.

**Additional file 5.** Coding Sequence of Irak1 and Traf6 Genes.

**Additional file 6.** Identification of hUCMSC, primary astrocyte and hUCMSC-derived exosomes. A hUCMSCs were identified by the stem cell makers (CD73, CD90 and CD105) and nonstem cell markers (CD45, CD34 and HLA-DR) expression using flow cytometry. B Primary astrocytes were characterized by immunostaining of GFAP, S100β, Vimentin and morphology in bright filed. C The morphology of exosomes detected by transmission electron microscopy. Scale bar = 200 nm. D Diameters and concentrations of exosomes analyzed by the nanoparticle tracking method. E Representative images of western blots to assess CD9, CD63 and β-actin expression in hUCMSCs and exosomes. F Uptake of the red fluorescence dye PKH-26 labeled exosomes into primary astrocytes. hUCMSCs human umbilical cord mesenchymal stem cells, EXO exosome, GFAP glial fibrillary acidic protein.

**Additional file 7.** A Successful delivery of PKH-26 labeled exosomes to the spinal cord lesion. Scale bar = 50 nm. B The successful delivery of miR-146a-5p to the spinal cord lesion.

**Additional file 8.** The other 4 abundant miRNAs mimics’ effects on mRNA expression of neurotoxic astrocyte markers. Quantitative analysis of mRNA expression of C3 and Lcn2 in astrocytes, neurotoxic astrocytes and neurotoxic astrocytes treated with 50 nM mimics of miR-21-5p(a), miR-22-3p(b), miR-24-3p(c), miR-26a-5p(d) by RT-QPCR. Data above are represented as mean ± SD. *, p < 0.05; **, p < 0.01; #, p>0.05. C3 complement c3, Lcn2 lipocalin-2.

**Additional file 9.** Detailed region where the representative images of Figure 2A selected from. LC, lesion cord.

**Additional file 10.** Detailed region where the representative images of Figure 5A selected from. LC, lesion cord.

## Data Availability

Not applicable.

## Abbreviations

C1q: Complement c1q
C3: Complement c3
CM: Conditioned medium
CM-Mix: CM obtained from neurotoxic astrocytes culture
CM-Mix + ExoN: CM from culture of neurotoxic astrocytes treated with exosomes
CM-Mix + ExoOE: CM from culture of neurotoxic astrocytes treated with miR-146a-5p-modified exosomes
CM-Mix + OE(Irak1&Traf6): CM from culture of neurotoxic astrocytes overexpressing Irak1 and Traf6
Exo: Exosomes
ExoN: Normal exosomes
ExoOE: MiR-146a-5p-modified Exosomes
HE: Hematoxylin and eosin
hUCMSCs: Human umbilical cord mesenchymal stem cell
IRAK1: Interleukin 1 receptor associated kinase 1
MSC-Exo: MSC-derived exosomes
NGF: Neuronal growth factor
OE(Irak1&Traf6): Overexpression of genes Irak1 and Traf6
PC12 cell: Pheochromocytoma cell (rat)
PDTC: Ammonium pyrrolidine dithiocarbamate
PVDF: Polyvinylidene difluoride
RT-QPCR: Quantitative real-time polymerase chain reaction
SCI: Spinal cord injury
SD: Standard difference
TPM: Transcripts per million
TRAF6: Tumor necrosis factor receptor-associated factor 6

## References

[1] McDonald JW, Sadowsky C (2002). Spinal-cord injury. Lancet.

[2] Tator CH, Fehlings MG (1991). Review of the secondary injury theory of acute spinal cord trauma with emphasis on vascular mechanisms. J Neurosurg.

[3] Norenberg MD, Smith J, Marcillo A (2004). The pathology of human spinal cord injury: defining the problems. J Neurotrauma.

[4] Fleming JC, Norenberg MD, Ramsay DA (2006). The cellular inflammatory response in human spinal cords after injury. Brain.

[5] Sofroniew MV, Vinters HV (2010). Astrocytes: biology and pathology. Acta Neuropathol.

[6] Liddelow SA, Barres BA (2017). Reactive astrocytes: production, function, and therapeutic potential. Immunity.

[7] Wanner IB, Anderson MA, Song B (2013). Glial scar borders are formed by newly proliferated, elongated astrocytes that interact to corral inflammatory and fibrotic cells via STAT3-dependent mechanisms after spinal cord injury. J Neurosci.

[8] Anderson MA, Burda JE, Ren Y (2016). Astrocyte scar formation aids central nervous system axon regeneration. Nature.

[9] Liddelow SA, Guttenplan KA, Clarke LE (2017). Neurotoxic reactive astrocytes are induced by activated microglia. Nature.

[10] van Niel G, D'Angelo G, Raposo G (2018). Shedding light on the cell biology of extracellular vesicles. Nat Rev Mol Cell Biol.

[11] Huang JH, Yin XM, Xu Y (2017). Systemic administration of exosomes released from mesenchymal stromal cells attenuates apoptosis, inflammation, and promotes angiogenesis after spinal cord injury in rats. J Neurotrauma.

[12] Li Y, Yang YY, Ren JL (2017). Exosomes secreted by stem cells from human exfoliated deciduous teeth contribute to functional recovery after traumatic brain injury by shifting microglia M1/M2 polarization in rats. Stem Cell Res Ther.

[13] Pirisinu M, Pham TC, Zhang DX (2022). Extracellular vesicles as natural therapeutic agents and innate drug delivery systems for cancer treatment: Recent advances, current obstacles, and challenges for clinical translation. Semin Cancer Biol.

[14] Elsharkasy OM, Nordin JZ, Hagey DW (2020). Extracellular vesicles as drug delivery systems: Why and how?. Adv Drug Deliv Rev.

[15] Lamichhane TN, Jeyaram A, Patel DB (2016). Oncogene knockdown via active loading of small rnas into extracellular vesicles by sonication. Cell Mol Bioeng.

[16] Schulz-Siegmund M, Aigner A (2021). Nucleic acid delivery with extracellular vesicles. Adv Drug Deliv Rev.

[17] Wang Y, Lai X, Wu D (2021). Umbilical mesenchymal stem cell-derived exosomes facilitate spinal cord functional recovery through the miR-199a-3p/145-5p-mediated NGF/TrkA signaling pathway in rats. Stem Cell Res Ther.

[18] Yanez-Mo M, Siljander PR, Andreu Z (2015). Biological properties of extracellular vesicles and their physiological functions. J Extracell Vesicles.

[19] Labbaye C, Testa U (2012). The emerging role of MIR-146A in the control of hematopoiesis, immune function and cancer. J Hematol Oncol.

[20] Saba R, Sorensen DL, Booth SA (2014). MicroRNA-146a: A dominant, negative regulator of the innate immune response. Front Immunol.

[21] Burnett JC, Rossi JJ (2012). RNA-based therapeutics: current progress and future prospects. Chem Biol.

[22] Lou G, Chen L, Xia C (2020). MiR-199a-modified exosomes from adipose tissue-derived mesenchymal stem cells improve hepatocellular carcinoma chemosensitivity through mTOR pathway. J Exp Clin Cancer Res.

[23] Qian D, Li L, Rong Y (2019). Blocking notch signal pathway suppresses the activation of neurotoxic A1 astrocytes after spinal cord injury. Cell Cycle.

[24] Lian H, Yang L, Cole A (2015). NFkappaB-activated astroglial release of complement C3 compromises neuronal morphology and function associated with Alzheimer's disease. Neuron.

[25] Taganov KD, Boldin MP, Chang KJ (2006). NF-kappaB-dependent induction of microRNA miR-146, an inhibitor targeted to signaling proteins of innate immune responses. Proc Natl Acad Sci U S A.

