# Supplementary material for: miR-146a-5p-modified hUCMSC-derived exosomes facilitate spinal cord function recovery by targeting neurotoxic astrocytes
Source: Stem Cell Res Ther. 2022 Sep 30;13:487. doi: 10.1186/s13287-022-03116-3 (PMC9524140; doi:10.1186/s13287-022-03116-3)
Supplement: Supplementary file 1 — Additional file 1. Animal protocol. [file 13287_2022_3116_MOESM1_ESM.docx]

| **Group**  **Day** | Sham (n=3) | SCI (n=32) | SCI+ExoN (n=33) | SCI+Exo-OE (n=32) |
| --- | --- | --- | --- | --- |
| 1 | NA | BBB score(n=10) | Exosome tracking(n=1); BBB score(n=10) | BBB score(n=10) |
| 3 | NA | QPCR(n=3);  BBB score(n=10) | QPCR(n=3);  BBB score(n=10) | QPCR(n=3);  BBB score(n=10) |
| 3 | IF(n=3) | IF, Tunnel staining(n=7) | IF, Tunnel staining(n=7) | IF, Tunnel staining(n=4) |
| 5 | NA | IF(n=4);  BBB score(n=10) | IF(n=4);  BBB score(n=10) | IF(n=4);  BBB score(n=10) |
| 7 | NA | IF(n=4);  BBB score(n=10) | IF(n=4);  BBB score(n=10) | IF(n=4);  BBB score(n=10) |
| 14 | NA | IF(n=4);  BBB score(n=10) | IF(n=4);  BBB score(n=10) | IF(n=4);  BBB score(n=10) |
| 28 | NA | BBB score, MRI, Foot print, IF, HE,  n=10 | BBB score, MRI, Foot print, IF, HE,  n=10 | BBB score, MRI, Foot print, IF, HE,  n=10 |

**Additional file 4. Animal protocol**

BBB, Basso, Beatlie, Bresnahan; ExoN, normal exosomes; ExoOE, exosomes with the overexpression of miR-146a-5p; HE, hematoxylin and eosin; IF, immunofluorescence; MRI, magnetic resonance image; NA, not available. SCI, spinal cord injury;
