# Supplementary material for: miR-146a-5p-modified hUCMSC-derived exosomes facilitate spinal cord function recovery by targeting neurotoxic astrocytes
Source: Stem Cell Res Ther. 2022 Sep 30;13:487. doi: 10.1186/s13287-022-03116-3 (PMC9524140; doi:10.1186/s13287-022-03116-3)
Supplement: Supplementary file 2 — Additional file 2. Supplementary methods and materials. [file 13287_2022_3116_MOESM2_ESM.docx]

**Animal experimental protocol**

All animal procedures were performed under the general anesthesia with 0.1% pentobarbital with 0.01 mL/g. Laminectomy of single vertebrae leaving the dura intact were performed to expose the spinal cord. An artery clamp (35 g for 30 s) was applied to compress the spinal cord at T10 level (3 mm depth for 30s). Successful spinal cord injury models would have following signs: flicking of bilateral hind limbs, swaying of the tail, hemorrhage and edema in injury site. A single laminectomy was performed in sham group without injury to spinal cord. Rats subjected to spinal cord injury were followed 200 μg (in 100 μl PBS) exosomes ExoN (normal) or ExoOE (with overexpression of miR-146a-5p) or equal volume PBS injection through the tail vein at the time of 30 min, 1 day, 2 days, 3 days post-injury. Massage of the bladder is used to help urite in two weeks post injury and 10^5^ IU penicillin would be used to anti-inflammation if it is necessary.

**BBB rating scale**

Neurological function was evaluated at 1, 3, 5, 7, 14, 21and 28 days post-injury (DPI) using the BBB score scale by two independent researchers who were blinded to groups in open field. Locomotor scores of each rat were recorded respectively. Foot prints analysis was also performed to evaluated the hind limbs locomotor function. The hindlimbs of the rat were dipped by black dyes. The stride lengths and widths were measured and analyzed when they ran at a straight line.

**Magnetic Resonance Imaging (MRI)**

Magnetic resonance imaging (MRI) was conducted at 28 days-post injury using a 1.5T MRI scanner (GE Signa Creator, USA). Rats were anesthetized with pentobarbital and fixated on animal used coil. Sagittal and axial T2-weighted images were scanned using FSE (fast spin echo) and FRFSE (fast recovery FSE) sequence. The slice thickness is 1 mm.

**Tunnel staining**

Rats were sacrificed by CO_2_ suffocation and 4% paraformaldehyde was perfused via ventriculus sinister. Then 1.5 cm long spine tissue was removed intactly around the injured epicenter and embedded into paraffin. After deparaffinization and rehydration, a 5 μm thickness longitudinal section were stained using the Tunnel detection kit (Servicebio, Wuhan, China) following the manufacturer’s instructions. The section was scanned by the [laser](javascript:;) [scanning](javascript:;) [confocal](javascript:;) [microscope](javascript:;) (Leica, Heidelberg, Germany).

**HE staining**

After deparaffinization and rehydration, a 5um thickness longitudinal section were stained using the hematoxylin and eosin solution. The slice then scanned by the laser scanning confocal microscope.

**Immunofluorescence**

**Cell**

Cells were firstly washed by phosphate buffer solution (PBS) and fixed by 4% paraformaldehyde for 30 min. Tris Buffered Saline Tween (TBST) containing 5% bovine serum albumin (BSA) was used to blocked the cell sample. 0.3% Triton X-100 was used for permeabilization. Primary antibodies against GFAP, C3, S100β, p-p65 (Abcam, Cambridge, UK), Lcn2(Biorbyt, Cambridge, UK) and Vimentin(CST, Boston, MA, USA) in blocking solution were incubated with the cell sample overnight at 4 ℃。

Then, fluorescein-conjugated secondary antibodies (Thermo Fisher, Boston, MA, USA) were used to hybridize primary antibodies. 4`,6-diamidino-2-phenylindole dihydrochloride (DAPI; Servicebio) was used for nuclear staining. Images were taken by laser scanning confocal microscopy.

**Tissue**

After deparaffinization and rehydration, spinal sections were blocked with 5% BSA and permeabilized in 0.5% Triton X-100. Then sections were incubated overnight at 4℃ with the following primary antibodies: anti-C3, anti-GFAP, anti-NeuN (Abcam, Cambridge, UK), anti-MAP2 (Servicebio, Wuhan, China) followed by fluorescein-conjugated secondary antibodies (Thermo Fisher, Boston, MA, USA). 4`,6-diamidino-2-phenylindole dihydrochloride (DAPI; Servicebio) was used for nuclear staining. Images were taken by laser scanning confocal microscopy.

**MSC characterization**

hUCMSCs purchased from Guangzhou Selera Stem Cell Technology Co., Ltd were characterized by surface markers using flow cytometry (BD Biosciences, Franklin Lakes, New Jersey, USA). Antibodies conjugated with fluorescein, including antibodies against CD73, CD90, CD105, CD34, CD45 and HLA-DR (Biolegend, San Diego, USA) were used to detect surface markers. Data were exported and analyzed using FlowJo software 10.0 (Stanford University, Palo Alto, USA).

**Exosome labeling**

Exosomes derived from hUCMSCs were labeled with PKH26 dye (Sigma-Aldrich, St. Louis, MO, USA) according to the manufactures’ instruction. Then the PKH26 solution with exosomes were suspended to 20 ml with PBS and re-ultracentrifuged (4°C, 100, 000 g for 60 min). Then the PKH26 labeled exosome were re-suspended in sterile PBS and stored at 4℃ for downstream experiment.

**Astrocyte isolation**

Methods for astrocyte isolation from spinal cord have been previous described[1]. Primary astrocytes were isolated from spinal cord of 2 weeks-old female rats. Remove the spinal cord from the spine column using a dorsal approach and place in cold Hank's Balanced Salt Solution (HBSS; Gibco, China). Carefully remove the meninges and blood vessels. Transfer the tissue to 35 mm dish and chop the tissue finely using a tissue scissor to generate a tissue slurry. Add 3 ml of 0.15% trypsin to the tissue slurry and incubate in 37 ℃ incubator with gentle shaking every 5 min to ensure spinal cord tissue remains suspended for 15 min. After incubation, add 3 ml Dulbecco's modified essential medium (DMEM; Gibco, China) with 10% fetal bovine serum (FBS; Gibco, Australia) to the medium. Triturate large tissue with pipette and taking care not to introduce air bubbles into the medium. Aspirate and filter the solution through a 100 μm nylon mesh filter into a 15 ml tube. Centrifuge at 1000 rpm for 5 min. Aspirate off the medium, resuspend the cells in DMEM with 10% FBS and plate the cells in a T75 flask for 1h to further remove the fibroblast. Then transfer the culture medium to a new T75 flask that has been previously coated with poly- L -lysine and incubate in incubator at 37℃, 5% CO_2_. Change the media every 2-3 days. After the cell reach 100% confluency, shake the flasks at 200 rpm for 6 h to remove the loosely adherent neurons and glia. Passage 3-5 primary astrocyte were used to further experiments.

**Culture medium collection**

Primary astrocytes reached 80-90% confluency were treated with IL-1α 3 ng/mL, TNF-α 30 ng/mL and C1q 400 ng/mL (Novoprotein, Shanghai, China) to induce neurotoxic astrocyte. Conditioned medium of astrocytes received different treatment was collected and concentrated with a 30 kDa Amicon Ultra-15 Centrifugal Filter Units (Millipore, UFC903024) until approximately 30-50× concentrated. BCA assay was performed to determine total protein concentration. 10-100 μg/ml total protein was added to culture of PC12 cells.

**Cell proliferation assay**

Cell Counting Kit-8 (Beyotime, Shanghai, China) was used to detect the PC12 cells viability. The absorbance of media at 450 nm was measured using a spectrophotometer (Biotek, Winooski, VT, USA). Cell viability was calculated via the followed formula: cell viability=Experiment (OD)-Blank (OD)/Control (OD)-Blank (OD).

**Neurite length assay**

PC12 cells were plated in a 6-well plate at a proper density. Culture medium (CM) with 1% FBS and 100 ng/ml neuronal growth factor (NGF; Proteintech, Chicago, Illinois, USA) was used to stimulate the neurite growth of PC12 for 7 days and was replaced every two days. At the seventh day of stimulation, CM previously collected from astrocytes received administration of Mix (IL-1α 3 ng/mL, TNF-α 30 ng/mL, and C1q 400 ng/mL, Novoprotein, Shanghai, China), Mix+ExoN, Mix+ExoOE, Mix+ExoOE+OE(Traf6&Irak1) was added to the medium for additional 24 hours culture. Three photographs were casually taken in 400x magnification in each group. Thirty cells with the longest neurites were included to calculate the mean neurite length which was analyzed by ImageJ (National Institute of Health, Maryland, USA).

**Transfection of Plasmids, miRNAs and siRNA**

Plasmids containing cDNA clones of Traf6 or Irak1 genes were constructed and purchased from IGEbio Co., Ltd. (Guangzhou, China). cDNA fragments were verified by DNA sequencing, RT-QPCR and electrophoresis and cloned into pCDNA3.1(+) vector (Life Technologies, Boston, MA, USA). miRNA sequence of miR-146a-5p were obtained from TargetScan Human 7.2. Mimics and miRDB.org. Inhibitors of miRNA were synthesized by RiboBio Co., Ltd. (Guangzhou, China). All plasmids, mimics and inhibitors were transfected by using Lipo-3000 reagent (Gibco, Carlsbad, California, USA). All sequences information used in this experiment were provided in **Additional file 6-7**.

**Luciferase reporter gene assay**

The wild type (WT) or mutant (MUT) 3'-UTR fragments of Traf6 mRNA and Irak1 mRNA were respectively cloned into psiCHECK-2 Dual Luciferase miRNA Target Expression Vector. The plasmids, miR-146a-5p mimics, inhibitors and negative control were transfected into HEK293 cells respectively by using lipo-3000 reagent. Luciferase activity was detected by Dual-Luciferase Reporter Assay System (Promega, Madison, Wisconsin, USA).

**RT-QPCR**

Total RNA of cells, spinal cord tissue and exosomes were extracted by using the Trizol reagent (Invitrogen, Carlsbad, California, USA). Concentration of total RNA was detected by NanoDrop (Thermo Fisher, Boston, MA, USA). cDNA of target genes was synthesized by reverse transcription using the StarScript II First-strand cDNA Synthesis Kit-II (Genstar, Beijing, China). Specific designed primers were used for reverse transcription of miRNAs and U6 by stem loop method. All primers were synthesized and purchased from RiboBio Co.; Ltd. (Guangzhou, China). Quantitative reverse transcription polymerase chain reactions (RT-QPCR) were carried out by using the RealStar Green Fast Mixture (with ROX II) (Gene Star, Beijing, China). β-actin and U6 were used as an endogenous control. Results were analyzed using the 2^-ΔΔCT^ method. The primer sequences are **provided in additional file 8.**

**Western blot**

Proteins of cells, spinal cord tissue and exosomes were extracted by using radioimmunoprecipitation assay (RIPA) buffer (Beyotime, Shanghai, China) with 1% Protease/Phosphatase Inhibitor Cocktail (CST, Boston, MA, USA) and quantified with BCA Kit. Equal amounts of protein were loaded into the gel and separated with SDS-PAGE, transferred to PVDF membranes (EMD Millipore Corp., Burlington, MA), blocked with 5%BSA and incubated with primary antibodies overnight at 4℃. Primary antibodies as followed: C3(Abcam, Cambridge, UK), Lcn2(Biorbyt, Cambridge, UK), P65(Abcam, Cambridge, UK), p-P65(Abcam, Cambridge, UK), Ikb (Abcam, Cambridge, UK), p-Ikb (CST, Boston, MA, USA), Traf6 (Affbiotech, Changzhou, China), Irak1(Affbiotech, Changzhou, China), beta-actin (CST, Boston, MA, USA), CD9(Abcam, Cambridge, UK), CD63(Abcam, Cambridge, UK), Histone (Affbiotech, Changzhou, China). Membranes then were incubated with secondary antibodies conjugated with horseradish peroxidase (HRP) for 1h at room temperature. Protein bands were visualized using enhanced chemiluminescence (ECL; Thermo Fisher, Boston, MA, USA) and the intensity of protein was analyzed using ImageJ software.

1. Kerstetter, A.E. and R.H. Miller, *Isolation and culture of spinal cord astrocytes.* Methods Mol Biol, 2012. **814**: p. 93-104.
