# Supplementary material for: miR-146a-5p-modified hUCMSC-derived exosomes facilitate spinal cord function recovery by targeting neurotoxic astrocytes
Source: Stem Cell Res Ther. 2022 Sep 30;13:487. doi: 10.1186/s13287-022-03116-3 (PMC9524140; doi:10.1186/s13287-022-03116-3)
Supplement: Supplementary file 3 — Additional file 3. Primers of target genes. [file 13287_2022_3116_MOESM3_ESM.docx]

| **Gene** | **Forward primer (5’-3’)** | **Reverse primer (5’-3’)** |
| --- | --- | --- |
| **C3** | AATTTATACCTTCCTTCCGCCT | GGAGTCCTTCACATCCACCC |
| **Lcn2** | ATTGACAACTGAACAGACGGTGA | TGGCAACAGGAAAGATGGAG |
| **TNFα** | CCCAGACCCTCACACTCAGAT | CTTGGTGGTTTGCTACGACG |
| **IL1β** | CCTCGTGCTGTCTGACCCAT | GTCGTTGCTTGTCTCTCCTTGTA |
| **IL4** | CACCTTGCTGTCACCCTGTTC | CTCTCTCAGAGGGCTGTCGTTAC |
| **IL6** | AGTCAACTCCATCTGCCCTTC | GTCTGTTGTGGGTGGTATCCTC |
| **IL10** | ACTGCTATGTTGCCTGCTCTTAC | GCATGTGGGTCTGGCTGACT |
| **Traf6** | GAATCACTTGGCACGGCACT | GAGAGGTTAACATTATGAACAGCCT |
| **Irak1** | AAGCAGAGCACCTAAGGGATTG | GCCAGCTCCACCATAGAAACAG |
| **β-actin** | TCAAGATCATTGCTCCTCCTGAG | ACATCTGCTGGAAGGTGGACA |

**Additional file 8. Primers of target genes**
