# Supplementary material for: miR-146a-5p-modified hUCMSC-derived exosomes facilitate spinal cord function recovery by targeting neurotoxic astrocytes
Source: Stem Cell Res Ther. 2022 Sep 30;13:487. doi: 10.1186/s13287-022-03116-3 (PMC9524140; doi:10.1186/s13287-022-03116-3)
Supplement: Supplementary file 4 — Additional file 4. miRNA sequences. [file 13287_2022_3116_MOESM4_ESM.docx]

| **Item** | **Sequence (5’-3’)** |
| --- | --- |
| **miR-146a-5p** |  |
| Mimic | UGAGAACUGAAUUCCAUGGGUU |
| Inhibitor | AACCCAUGGAAUUCAGUUCUCA |
| **miR-21-5p** |  |
| Mimic | UAGCUUAUCAGACUGAUGUUGA |
| **miR-22-3p** |  |
| Mimic | AAGCUGCCAGUUGAAGAACUGU |
| **miR-24-3p** |  |
| Mimic | UGGCUCAGUUCAGCAGGAACAG |
| **miR-26a-5p** |  |
| Mimic | UUCAAGUAAUCCAGGAUAGGCU |
| **Mimic NC** |  |
|  | UUUGUACUACACAAAAGUACUG |
| **Inhibitor NC** |  |
|  | CAGUACUUUUGUGUAGUACAAA |

**Additional file 6. miRNA sequences**
