# Supplementary material for: miR-146a-5p-modified hUCMSC-derived exosomes facilitate spinal cord function recovery by targeting neurotoxic astrocytes
Source: Stem Cell Res Ther. 2022 Sep 30;13:487. doi: 10.1186/s13287-022-03116-3 (PMC9524140; doi:10.1186/s13287-022-03116-3)
Supplement: Supplementary file 5 — Additional file 5. Coding Sequence of Irak1 and Traf6 Genes. [file 13287_2022_3116_MOESM5_ESM.docx]

**Additional file 7. Coding Sequence of Cbl and Cblb Genes**

| **Gene** | **Sequence** |
| --- | --- |
| **Traf6**  **[NM_001107754.2 (complete CDS)]** | atgagtctct taaactgtga aaacagctgt gcgtccagcc agtcttcaag  241 cgactgctgt gctgccatgg ccaactcctg cagtgctgcc atgaaagatg acagtgtgag  301 tggctgtgtc agcacgggga acctgtccag ctccttcatg gaggagatcc agggatatga  361 tgtggagttt gacccacctt tggaaagcaa gtatgagtgc cccatctgct tgatggcttt  421 acgggaagca gtgcaaacac catgtggcca caggttctgc aaagcctgca tcaccaagtc  481 cataagggat gcaggtcaca agtgcccagt tgacaatgaa atactgctgg aaaatcaact  541 gtttcctgac aattttgcaa agcgagagat tctttccctg acggtaaagt gtccaaataa  601 aggctgtgtg caaaagatgg agctgagaca tctcgaggat catcaagtac attgtgaatt  661 cgctctagtg atttgtcccc aatgccaacg ttttttccaa aagtgccaga ttaataaaca  721 cattatcgag gattgtccca ggagacaggt ttcttgtgta aactgtgctg tgcccatgcc  781 gtatgaagag aaagagatcc acgatcaaag ctgtcctctg gcaaatatca tctgtgaata  841 ctgtggtaca atcctcataa gagaacagat gcctaatcat tatgatctag actgcccaac  901 agctccagtc ccctgcacat tcagtgtgtt tggctgtcac gaaaagatgc agaggaatca  961 cttggcacgg cacttgcaag agaacaccca gttgcacatg agactgttgg cccaggctgt  1021 tcataatgtt aacctctctt tgcggccatg cgatgcctcc tctccatccc ggggatgtcg  1081 tcctgaggac ccaaattatg aggaaacggt caaacagttg gaggggcgcc tagtaagaca  1141 ggaccatcaa atccgggagc tgaccgccaa aatggaaacg cagagcatgc atgtgagcga  1201 gctcaagcgg accattcgaa gcctcgagga caaagttgcc gagatggaag cacagcagtg  1261 taatggcatt tacatttgga agattggcaa ctttgggatg cacttgaaat cccaagaaga  1321 ggaaagacct gtggtcattc atagccctgg attctacaca ggcagacctg ggtacaagct  1381 gtgcatgcgc ctgcacctcc agctaccgac ggctcagcgc tgtgcaaact acatttccct  1441 ctttgtccac acaatgcaag gagagtatga cagccacctc ccctggccct tccagggtac  1501 aatacgcctc acgatccttg atcagtctga agcagtaata aggcaaaacc acgaagaggt  1561 catggatgct aagccagaac tgcttgcctt tcagcggccc accatcccac ggaaccccaa  1621 aggttttggc tatgtgacat tcatgcacct ggaagcctta agacagggaa ccttcatcaa  1681 ggatgatacg ttattagtgc gctgtgaagt ctctacccgc tttgacatgg ggggccttcg  1741 gaaggagggg ttccagccac ggagtactga tgcaggcgtg tag |
| **Irak1**  [**NM_001127555.1 (complete CDS)**] | atggccggg ggcccgggcc ccggggagcc tgtggttccc ggcgcccagc atttcttgta  241 cgaggtgcct ccctgggtta tgtgccgttt ctacaaagtg atggacgccc tggagcccgc  301 cgactggtgc cagttcgcgg ccttgatcgt gcgcgaccag acagagctgc ggctgtgtga  361 gcgctccgag cagcgcacgg ccagtgtcct gtggccctgg atcaaccgca acgcgcgtgt  421 ggctgacctc gttcacatcc tcacgcacct gcagctgctg cgtgcgcggg acatcattac  481 agcctggcac cctcctgcct ctgttctgcc cccaagcacc ggtgccccaa ggcccagcag  541 catctctgca ggctctgaga ctgaggactg gagcccccgg aaattgcagt cctctgcctc  601 caccttcctc tccccagctt ttccaggctc ccagacccat tctgagtcag agctcctcca  661 ggttccactc tctgattccc tcgggccacc actacaatct tcagccccct cctccatcaa  721 gccaagccca gagagcccag tgtctggcct ccaaagagct cgtccctccc cattttgctg  781 gcccttctgt gagatttccc aaggcacttg caacttctct gaagaactca ggattggaga  841 gggtggtttt gggtgtgtgt accgagcagt aatgagaaat actacatatg ctgtgaagag  901 actgaaggag gaggctgacc tagagtggac tgtggtgaaa cagagcttcc taacagaggt  961 ggaacagcta tcaaggtttc gtcacccaaa tatcgtggac tttgctggct actgtgcaga  1021 gagtggcttc tactgccttg tttatggctt cctgcccaat ggctccttag aggatcagct  1081 tcaccttcag acccaagcct gctccccact ttcctggcct caacgactgg acattcttct  1141 gggcacagcc cgggctattc agtttttaca tcaggacagc cccagcctta tccatggaga  1201 catcaagagt tctaatgtgc ttctggatga gagactgatg cccaagctgg gagactttgg  1261 tctggctcgt ttcagccgct ttgcaggggc caacccaagc cagagcagta ctgtggcccg  1321 gacttgcaca gttcgaggta ccctcgccta cctgcctgag gagtacatca agacaggccg  1381 gctggctgtg gacaccgata ccttcagctt tggggtggta atactggaga cccttgctgg  1441 tcagagggct gtgaggacac aaggtgcaaa aaccaaatat ctgaaagacc tgattgaaga  1501 tgaggctgaa gaggtgggag tgaccttaaa aagcacccag cctactttgt ggatgggtgt  1561 agccacggat gcttggactg ctccaattgc tgcccagatc tataagaagc acctggactc  1621 cagacctggg ccctgcccac cccagttggg cctggccctg gctcaactag cttgctgctg  1681 catgcaccgt cgggccaaga agaggccacc catgacccag gtatacaaga gactagaagg  1741 gcttcaggca gtacctccct ggaagctaga ggttgccggc catggctctc cttccccaca  1801 ggagaactcc tacatgtcca ccactggcag tgcccagagt ggggatgaac catggcagcc  1861 tctggtagtg accacaagag ctccagccca ggctgcccag caactccaga gaagtcccaa  1921 ccagccagtg gagagtgatg agagtgttcc tggcctctct gctaccctgc attcctggca  1981 cttgactcca gattcccacc caagccctgt gtccttcaga gaggctagct gtacacaagg  2041 aggcactacc agagaatcaa gtttgaggag tggcccaggc ttccagccta cgaccatgga  2101 aggctcatcg atgggcagtt catccctgct gtcatcagag ccaccacaga tcatcatcaa  2161 cccagcccga cagaagatgg tacaaaagct ggccctgtat gaagaggggg tcttggatag  2221 cctgcaactg ctgtcatcag gctttttccc aggcttggat ttagaacctg aaaagagaaa  2281 gagacctgaa gaaagtgatg aattccagag ctga |
