# Supplementary figures and images for: miR-146a-5p-modified hUCMSC-derived exosomes facilitate spinal cord function recovery by targeting neurotoxic astrocytes
Source: Stem Cell Res Ther. 2022 Sep 30;13:487. doi: 10.1186/s13287-022-03116-3 (PMC9524140; doi:10.1186/s13287-022-03116-3)

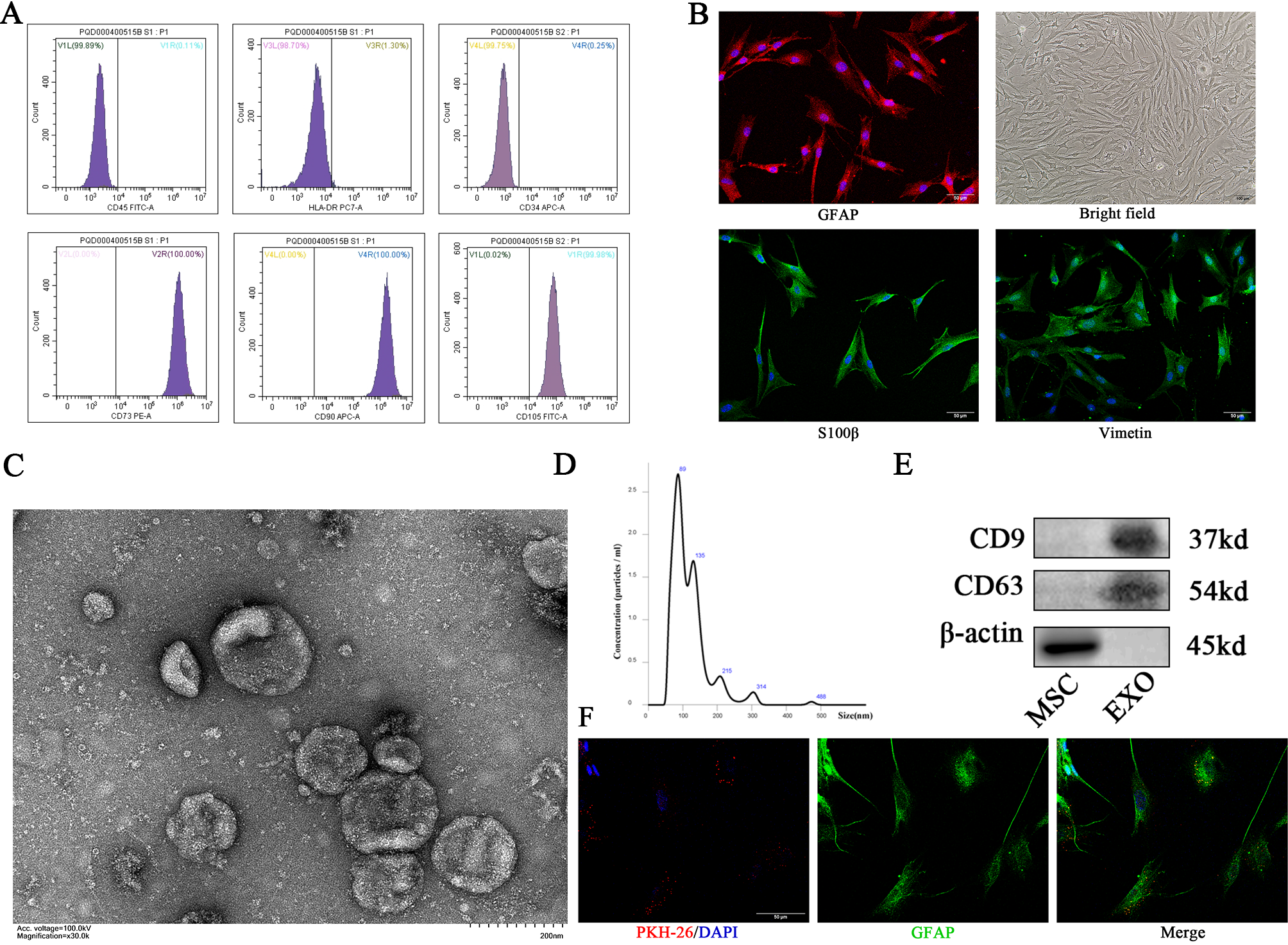

Supplement: Supplementary file 6 — Additional file 6. Identification of hUCMSC, primary astrocyte and hUCMSC-derived exosomes. A hUCMSCs were identified by the stem cell makers (CD73, CD90 and CD105) and nonstem cell markers (CD45, CD34 and HLA-DR) expression using flow cytometry. B Primary astrocytes were characterized by immunostaining of GFAP, S100β, Vimentin and morphology in bright filed. C The morphology of exosomes detected by transmission electron microscopy. Scale bar = 200 nm. D Diameters and concentrations of exosomes analyzed by the nanoparticle tracking method. E Representative images of western blots to assess CD9, CD63 and β-actin expression in hUCMSCs and exosomes. F Uptake of the red fluorescence dye PKH-26 labeled exosomes into primary astrocytes. hUCMSCs human umbilical cord mesenchymal stem cells, EXO exosome, GFAP glial fibrillary acidic protein. [file 13287_2022_3116_MOESM6_ESM.tif]

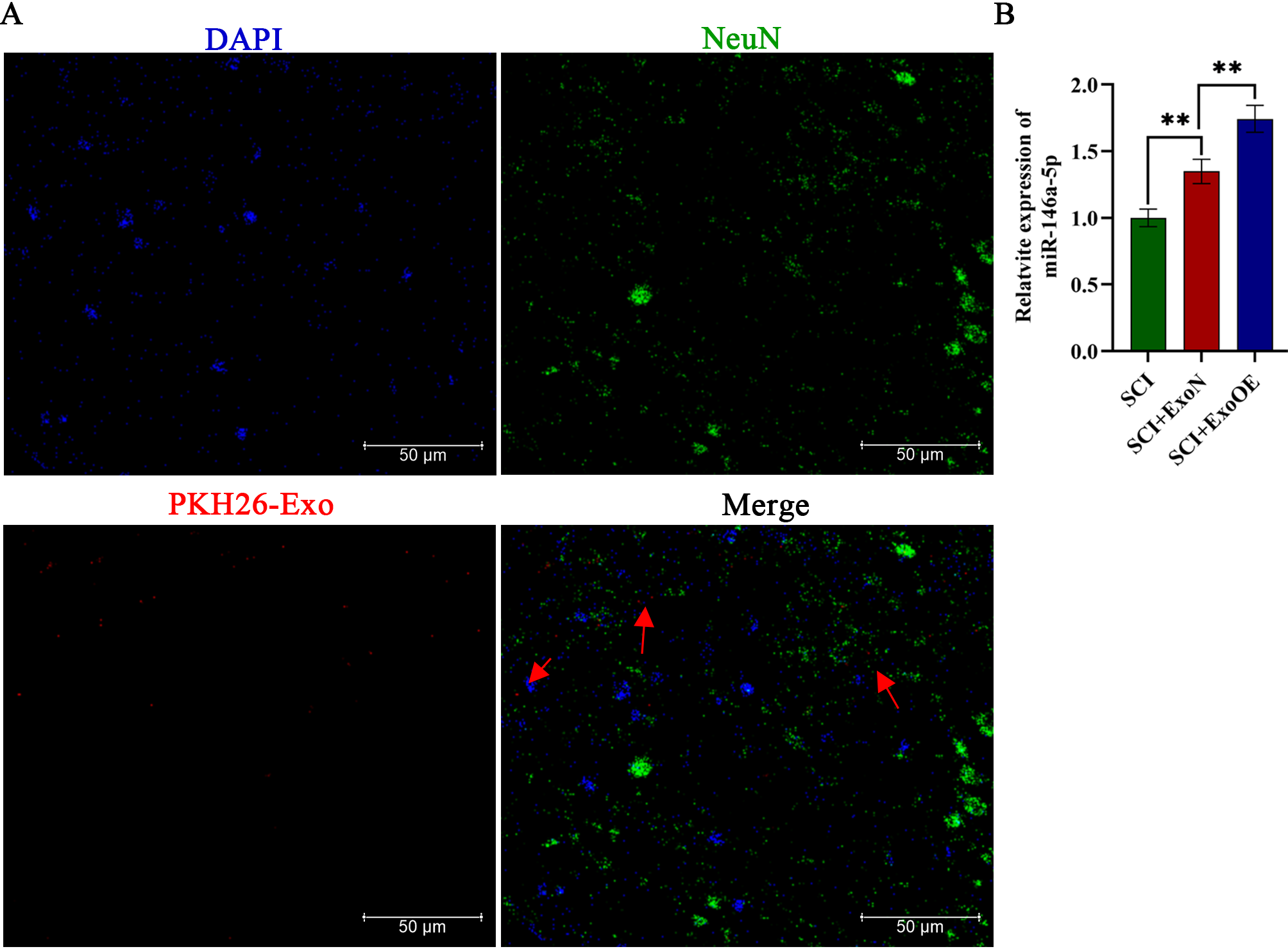

Supplement: Supplementary file 7 — Additional file 7. A Successful delivery of PKH-26 labeled exosomes to the spinal cord lesion. Scale bar = 50 nm. B The successful delivery of miR-146a-5p to the spinal cord lesion. [file 13287_2022_3116_MOESM7_ESM.tif]

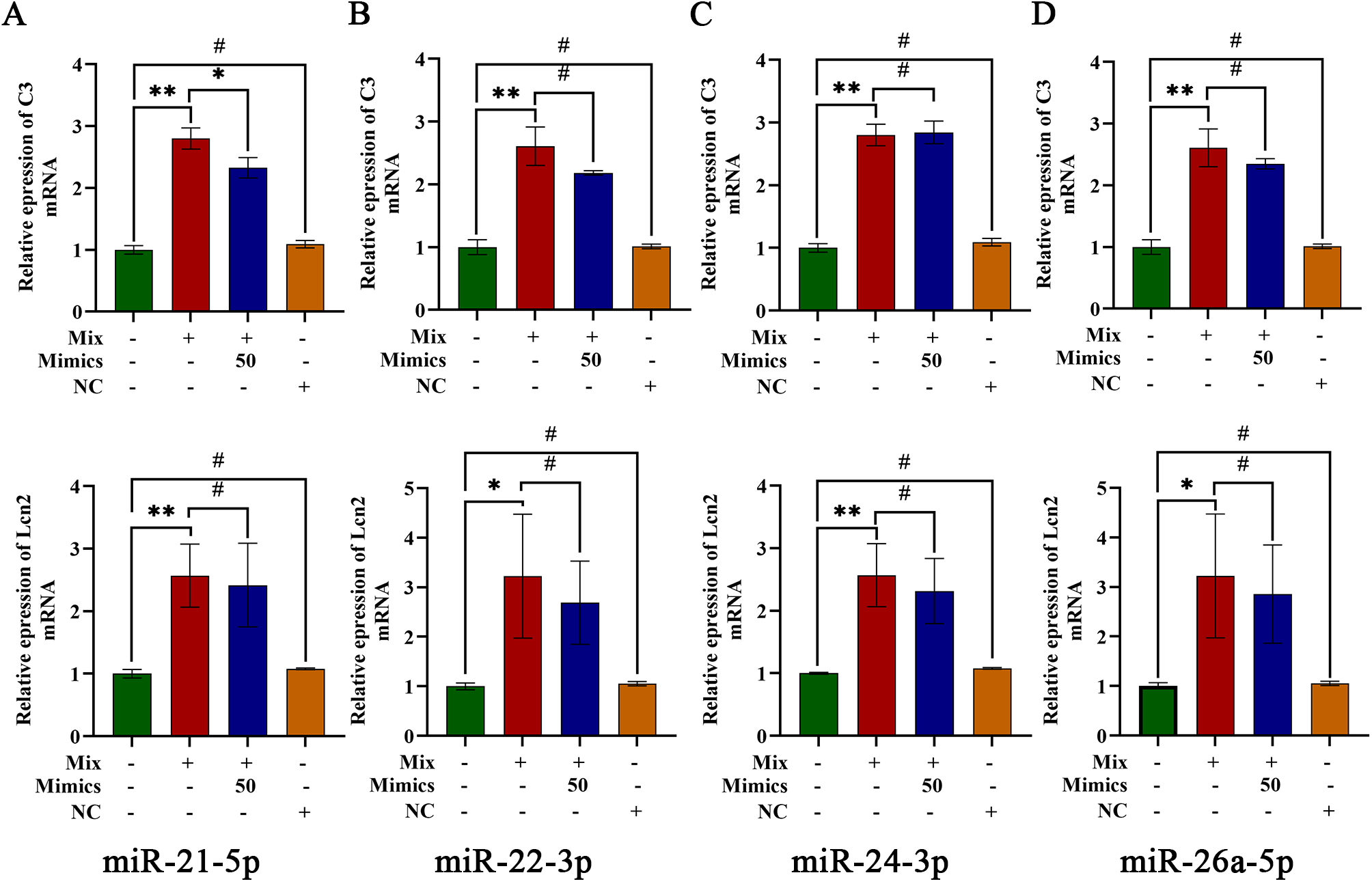

Supplement: Supplementary file 8 — Additional file 8. The other 4 abundant miRNAs mimics’ effects on mRNA expression of neurotoxic astrocyte markers. Quantitative analysis of mRNA expression of C3 and Lcn2 in astrocytes, neurotoxic astrocytes and neurotoxic astrocytes treated with 50 nM mimics of miR-21-5p(a), miR-22-3p(b), miR-24-3p(c), miR-26a-5p(d) by RT-QPCR. Data above are represented as mean ± SD. *, p < 0.05; **, p < 0.01; #, p>0.05. C3 complement c3, Lcn2 lipocalin-2. [file 13287_2022_3116_MOESM8_ESM.tif]

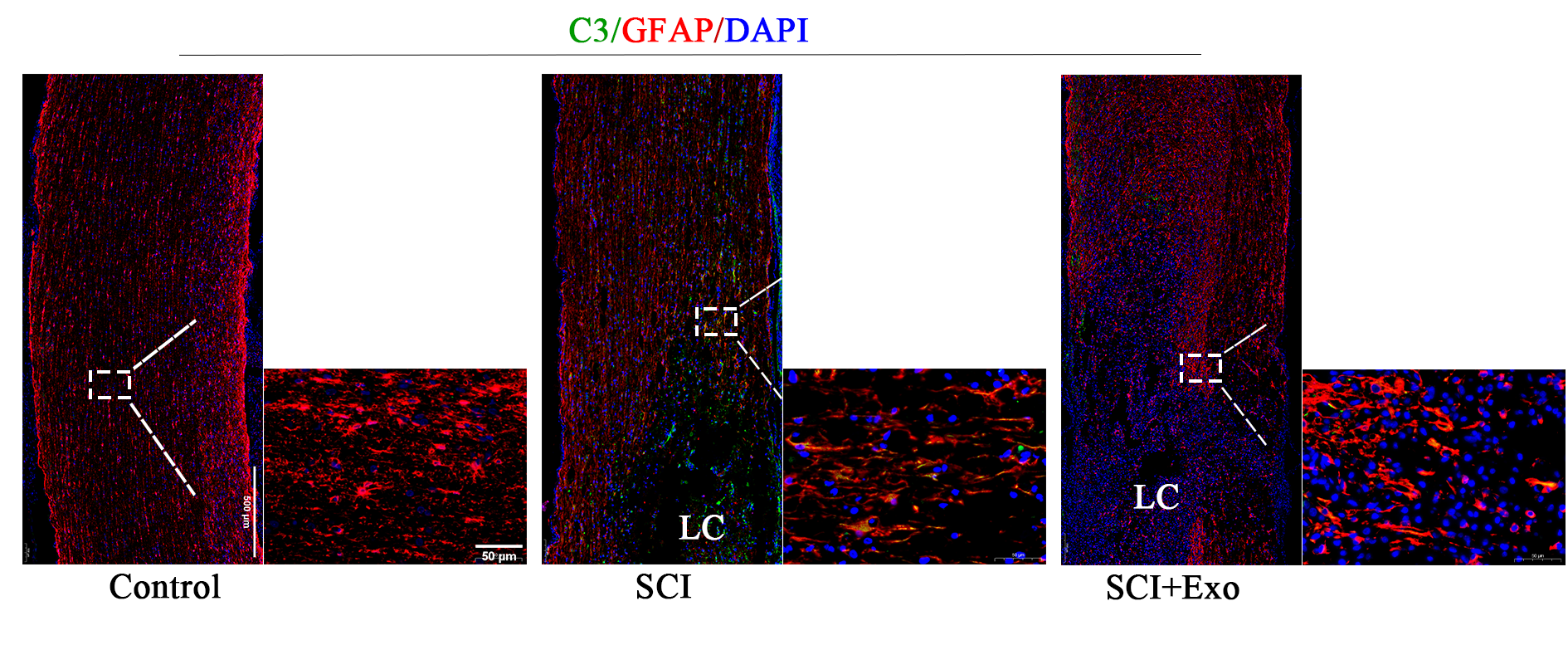

Supplement: Supplementary file 9 — Additional file 9. Detailed region where the representative images of Figure 2A selected from. LC, lesion cord. [file 13287_2022_3116_MOESM9_ESM.tif]

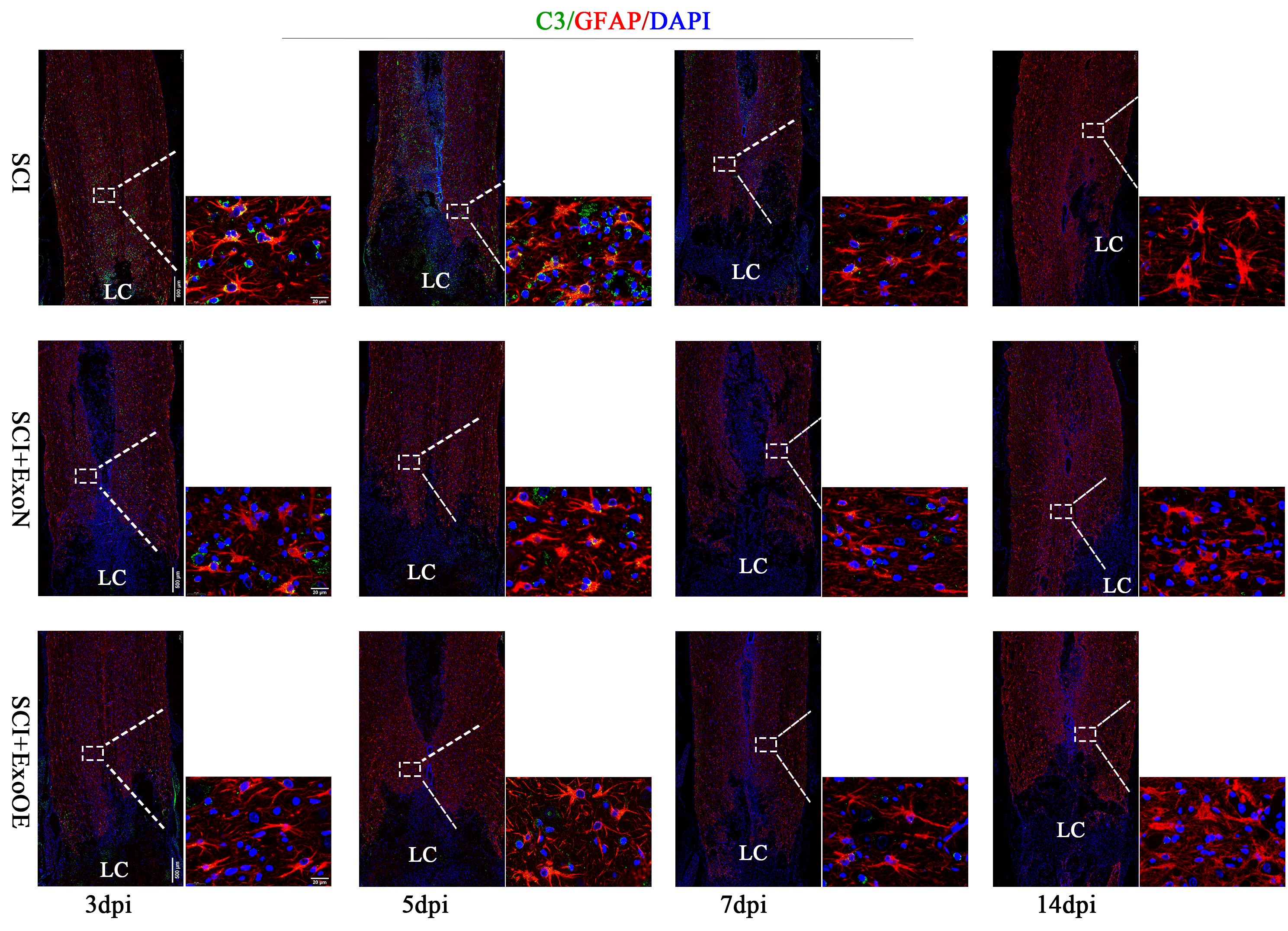

Supplement: Supplementary file 10 — Additional file 10. Detailed region where the representative images of Figure 5A selected from. LC, lesion cord. [file 13287_2022_3116_MOESM10_ESM.tif]
